# Weak-form inference for hybrid dynamical systems in ecology

**DOI:** 10.1098/rsif.2024.0376

**Published:** 2024-12-18

**Authors:** Daniel Messenger, Greg Dwyer, Vanja Dukic

**Affiliations:** ^1^ Department of Applied Mathematics, University of Colorado, Boulder, CO, USA; ^2^ Department of Ecology & Evolution, University of Chicago, Chicago, IL 60637, USA

**Keywords:** multi-scale model, hybrid systems, data-driven modelling, system identification, parameter estimation, WSINDy

## Abstract

Species subject to predation and environmental threats commonly exhibit variable periods of population boom and bust over long timescales. Understanding and predicting such behaviour, especially given the inherent heterogeneity and stochasticity of exogenous driving factors over short timescales, is an ongoing challenge. A modelling paradigm gaining popularity in the ecological sciences for such multi-scale effects is to couple short-term continuous dynamics to long-term discrete updates. We develop a data-driven method utilizing weak-form equation learning to extract such hybrid governing equations for population dynamics and to estimate the requisite parameters using sparse intermittent measurements of the discrete and continuous variables. The method produces a set of short-term continuous dynamical system equations parametrized by long-term variables, and long-term discrete equations parametrized by short-term variables, allowing direct assessment of interdependencies between the two timescales. We demonstrate the utility of the method on a variety of ecological scenarios and provide extensive tests using models previously derived for epizootics experienced by the North American spongy moth (*Lymantria dispar dispar*).

## 1. Introduction

The ability to estimate model parameters and select appropriate models based on data is of central interest in ecological applications but is often complicated by the presence of irregularly observed sparse observations [1]. Fortunately, the widespread availability of high-performance computing and the development of sophisticated nonlinear optimization algorithms have made it possible to fit a vast range of nonlinear ecological models to data [2]. Many applications of model-fitting have focused on human diseases, for which an extensive knowledge base makes it possible to write down at least basic models using only information from the literature [3]. A very large set of ecological problems, however, instead involve natural systems about which so little is known that there is often a great deal of uncertainty about the model structure [1].

In such cases, the standard approach is to write down a set of competing models, each comprising what is believed to be a reasonable model structure, to fit each model separately to the data and then to use statistical model selection to choose between the models [4–6]. Because in such cases, the models in question are typically based on well-studied ecological models from the literature, the use of model selection results in a model that has a well-understood connection to the biology of the system being studied. Model selection can then offer important insights into the biology of the system [7].

A very important disadvantage of the application of model selection to ecological problems, however, is that it typically requires the implementation of complex nonlinear fitting routines in high-performance computing environments, and this implementation must be repeated for each model under consideration [8]. Model selection in ecology therefore faces daunting computational obstacles when even moderate numbers of models are considered, which is probably why it has played only a modest role in the development of the field.

Here, we therefore propose a novel alternative approach based on weak-form equation learning [9–11] to accomplish the joint parameter estimation and model selection task, which we will jointly refer to throughout as ‘model identification’ in this work. By simultaneously comparing a very large number of models to data at once, our model identification algorithm is able to carry out model selection far more rapidly than the standard information-criterion model selection process. Moreover, our algorithm produces models that can very accurately reproduce data.

The models that we consider consist of host–pathogen models that allow for seasonality. Although classical host–pathogen models typically neglect seasonality [12], host–pathogen interactions in nature are often very strongly affected by seasonality [13]. Moreover, standard models typically incorporate seasonality in the form of sinusoidal variation in a model parameter, often host reproduction. But this model structure is deeply unrealistic for most host–pathogen systems.

For most host–pathogen systems, there is instead typically a period during which pathogen transmission exhausts or nearly exhausts the supply of available hosts. Transmission then falls to zero or nearly so until host reproduction replenishes the supply of susceptible hosts. As briefly summarized by Andreasen & Dwyer [14], this pattern occurs in host–pathogen systems for which hosts are all classes of vertebrates, most if not all terrestrial phyla of invertebrates, and many different plants, while the pathogens include viruses, bacteria and fungi. Our algorithm is customized to adapt to this specific structure by carrying out model identification using a combination of short-term disease dynamics and long-term population fluctuations.

We consider, in particular, the case of baculovirus pathogens of forest insects, which often drive long-period, large-amplitude cycles in the population densities of their insect hosts [15]. Because only larvae are susceptible to baculoviruses, virus epizootics (epidemics in animals) only occur during the larval season. Larvae that survive to adulthood reproduce, while the virus overwinters on the insect’s eggs. The epizootic is then restarted in the following spring when some larvae become infected as they hatch. Because many outbreaking host insects have only one generation per year [16], and because the larval period typically lasts for only two months [17], our models consist of continuous-time epizootic models that are nested inside discrete-generation host-reproduction/virus-overwintering models.

These multi-scale hybrid models of insect-pathogens were introduced in the ecological literature by Briggs & Godfray [18] and have been extended to include evolutionary change [17,19–21], variation in the foliage quality of insect host trees [22] and weather effects [7]. Hybrid models have been extensively applied to host–pathogen dynamics more generally [23–25] and to consumer–resource theory [26,27], sometimes drawing fundamentally different ecological implications compared with purely discrete or purely continuous models.

### 1.1. Inference and learning in dynamical systems

When the model system equations are of known form, the unknown system parameters and initial conditions are usually inferred from the data using statistical methods. However, when the model itself is unknown (in addition to having unknown parameters), the problem of statistical inference becomes much more difficult. We discuss both situations next.

#### 1.1.1. Parameter Inference

In spite of the availability of sophisticated optimization algorithms and high-performance computing, parameter estimation and inference (and inverse problems in general) in nonlinear dynamical systems remain a challenging problem [28]. Many of the existing parameter estimation methods in practice, such as nonlinear least squares and likelihood-based methods, still use optimization (cost) functions which are based on comparisons of data to the solution of the system. This results in iterative schemes that use a forward-solver-based optimization routine to minimize the nonlinear least squares residual at every iteration. Furthermore, the likelihood functions commonly resulting from nonlinear differential equations often exhibit multiple modes and ridges, with different combinations of parameter values yielding similar state values. In most scenarios, converging to globally optimal and unique parameter values can be challenging, requiring long run times and careful diagnostic monitoring [29–31].

Several alternative approaches have therefore been put forward in an attempt to reduce or altogether avoid using forward-based solvers, with varying levels of success: via likelihood-free sequential Monte Carlo methods [32], local approximations [33] and manifold-constrained Gaussian processes [34]. Sometimes a purely statistical approach (such as spline and kernel regression methods in [35]) is used instead to model a smooth temporal pattern. With the exception of the Gaussian-process-based approach in [34], none of the above methods are guaranteed to solve the underlying dynamical system; our weak-form approach in contrast guarantees that. Moreover, the Gaussian-process approach in [34] requires Markov chain Monte Carlo (MCMC) sampling to do so and thus it takes notably longer to produce results than the weak-form algorithm presented here.

#### 1.1.2. Model equation learning

As we described earlier, the standard approach to the model learning problems in ecology is to write down a small candidate set of models based on a general understanding of the organisms in question, and then to fit the parameters in each model as if that model was the true model. Statistical model selection is then performed using an information criterion (such as Akaike (AIC), Bayesian (BIC), Watanabe (WAIC), etc.) [6,7,17,36–39]. This process requires a great deal of knowledge of the biology and sizeable computational resources and is particularly challenging when the models under consideration have either high parameter dimensions or high-dimensional states, or both in the case of hierarchical and random differential equation models [22,39], or in the case of strong seasonality and multiple coupled models, like the discrete-continuous hybrid framework that we consider here.

Our approach is to instead write down very general expressions for the model structure, and then to use data to choose the actual model structure from a large library 𝕃 consisting of |𝕃| possible equation terms (where |𝕃| in our examples is allowed to reach 562 through adaptive expansion), resulting in $2^{|𝕃|}$ possible candidate models for consideration. Such large model spaces cannot be explored in their entirety, but instead rely on guided methods for sequential exploration, such as sequential thresholding [40,41]. The equation learning methodology presented here thus results in the identification of interpretable forms of dominant mechanisms governing a hybrid discrete-continuous dynamical system for population dynamics, among combinations of hundreds of possible linear and nonlinear terms in the library, without the need to separately fit different models to the data.

Previous methods for model equation learning for hybrid dynamics have generally handled parameter inference only after the final form of the model has been chosen. Such methods include combined sparse identification and clustering [42,43], neural networks [44,45] and online learning [46]. Ecological hybrid modelling calls for new tools, particularly to address complex dependencies between the discrete and continuous modelling stages. In addition, in contrast to engineering applications that are the focus of [42], in many ecological settings, the switching times between continuous stages are seasonal and therefore known *a priori*. This implies that many existing methods may be unnecessarily complex, providing non-optimal or inefficient estimators for ecological hybrid models.

Here, we develop a novel data-driven method that performs weak-form ‘equation-learning’ methodology simultaneously with parameter inference [9–11], in the context of seasonal hybrid dynamical systems. Our method can be seen as a weak-form extension of the sparse hybrid system identification method [42] with statistically sound parameter estimation and special adaptations for *seasonal* hybrid systems, where each ‘generation’ has a fixed length, a common setting in ecology. A core technique in the inference of seasonal hybrid systems is our novel employment of the sparse parametric modelling technique in [47], whereby we alternate between interpreting the discrete variables as parameters in the continuous dynamics, and interpreting the (final-time) continuous variables as parameters in the discrete dynamics. This method is made particularly effective by incorporating parameter inference as in [9], which accurately models noise in the data and thereby enables quantification of parameter uncertainty.

Our weak-form method is highly efficient as it does not employ forward-solvers during model identification, requiring of the order of 30 s on a modern laptop to complete (§4.2 describes performance measures for hardware specifications). Efficiency is granted by (i) employing the WENDy algorithm for parameter estimation (weak-form estimation of nonlinear dynamics), which was demonstrated in [9] to be orders of magnitude faster than nonlinear least squares-based methods, and by (ii) selecting models using sparse regression with adaptive library expansion instead of an exhaustive search. The latter enables consideration of a large set of models (of the order of $2^{|𝕃|}$, although efficient elimination of inaccurate models leads to explicit testing on only a small fraction of this total). Moreover, compared with other sparse equation learning methods that employ the strong form, weak-form sparse regression methods as employed here have high noise tolerances (e.g. [10,11]), which demonstrate that the noise level may be increased by at least an order of magnitude without sacrificing speed or accuracy). The method we introduce here also has relatively low data requirements. A sparse set of intermittent observations is sufficient to identify a coupled system of short-term continuous dynamics and long-term discrete dynamics, each a generalization of the annual epidemics and overwintering effects common to seasonal host–pathogen systems. This enables a variety of inference tasks common to ecology to be performed efficiently, such as prediction of future boom and bust cycles. In fact, as we will show in §4, data on 18 non-consecutive generations can enable accurate prediction for upwards of 40 generations with our method. The sampling *strategy*, however, does impact performance, as noted in previous SINDy-type studies [10,48,49], and we confirm in §4.6 that peak sampling, the approach most commonly conducted in field studies,^1^ is significantly more efficient than random sampling.

Our method learns a parametrization of the continuous-time model by the discrete-time variables (and vice versa) using the weak-form model learning algorithm WSINDy (weak-form sparse identification of nonlinear dynamics [10]), an extension of the SINDy algorithm [40] and is therefore a novel approach to modelling hybrid systems [52,53]. In this way, our method can be directly applied to general hybrid systems with known event times (i.e. *seasonality*), as arise in a variety of disciplines including epidemiology [12], endocrinology [54], oncology [55] and even security for cyberinfrastructure [56]. Furthermore, learning a system of differential equations using WSINDy, jointly with the recently developed weak-form parameter inference method WENDy [9], constitutes a novel approach to model learning (see §3.1 for an overview of WSINDy and WENDy). In addition, both WSINDy and WENDy are novel to ecology applications. WENDy has been shown to lead to a substantial improvement over both traditional nonlinear least-squares optimization [9] and Bayesian methods [34,57]. The resulting improvements in our ability to identify the dominant mechanisms in both short- and long-term modelling components have the potential to improve our understanding of the underlying ecology, to produce better model-based predictions and to better inform future control strategies. The weak-form equation-learning methods presented here therefore have the capability to allow model identification and inference for a broad class of dynamical systems that express some form of seasonality, and that therefore lie outside of the current capabilities of equation learning.

This article is organized as follows. In §2, we present more details on the insect-pathogen systems that motivate our seasonal hybrid models. In §3, we describe our proposed model identification method, including a high-level overview of our weak-form equation learning methodology in §3.1, and a description of the complete algorithm in §3.2. In §4, we perform a series of numerical experiments to assess the performance of the method (§§4.4 and 4.6), and in addition, we provide a detailed roadmap of how to interpret, build upon and utilize the results of the algorithm in practice to make predictions and quantify uncertainty (§4.5). A discussion is provided in §5. Finally, the appendix contains notation tables, as well as an additional example, analyses and algorithm pseudocode.

## 2. Motivation

In order to motivate the model class relevant to this paper, in §2.1, we review a simplified model for the dynamics of insect–baculovirus interactions, which will form the basis of our numerical experiments in §4. The general class of seasonal discrete-continuous models is presented in §2.2.

### 2.1. Seasonality in host–pathogen systems

Animal and plant host–pathogen systems commonly have a high degree of seasonality that depends on the timing of host reproduction and pathogen transmission (see [14] and references therein). In many animal hosts, notably invertebrates and amphibians, pathogen transmission can only occur during the larval stage, while in plants host transmission often only occurs during the growing season. Meanwhile, transmission in many bird and mammal hosts often ends because of a lack of susceptible hosts. In either type of host–pathogen interaction, long periods of disease transmission strongly reduce the susceptible host population, after which short periods of host reproduction replenish the susceptible host population. The ubiquitous effect of seasonality in ecological systems demands a robust modelling and inference framework, which we argue is met by hybrid discrete-continuous models and the inference method that we present here.

To introduce the hybrid discrete-continuous modelling framework, and to demonstrate its applicability to strongly seasonal ecological systems, we consider a model for insect–baculovirus dynamics [19]. As we described earlier, baculoviruses represent a large group of viral pathogens of arthropod hosts [58]; in many insects in particular, baculoviruses are transmitted when uninfected larvae that are feeding on the foliage of their host plant accidentally consume infectious occlusion bodies released from the cadavers of hosts killed by the virus [59]. Baculoviruses must thus kill their host to be transmitted [60]. For many species of forest insects, baculoviruses decimate outbreaking populations [50,61], and therefore appear to drive long-period, large-amplitude predator–prey type cycles in populations of their hosts [62]. The ability of mathematical host–pathogen models to reproduce these cycles provides support for this hypothesis [15,19].

Outbreaking forest insects typically have only one generation per year [16]. Because only larvae can become infected, and because host reproduction therefore occurs after each year’s viral epizootic is over, we first construct a continuous-time model of baculovirus dynamics during the larval period, using a modification of a standard susceptible-infected-recovered (SIR) model from theoretical epidemiology [12]. Let $S_{n}(t)$ denote the density of susceptible host larvae and $P_{n}(t)$ the density of baculovirus-infected cadavers in year n (the nth generation). We then have the following short-term epizootic model running from time t=0 to t=T (in days, relative to the start of the generation) in terms of ordinary differential equations:


(2.1) $$\left\{ \begin{array}{l} {\dot{S}}_{n}=-\bar{\nu }S_{n}P_{n}{\left(\frac{S_{n}(t)}{S_{n}(0)}\right)}^{V}\\ {\dot{P}}_{n}=\bar{\nu }S_{n}P_{n}{\left(\frac{S_{n}(t)}{S_{n}(0)}\right)}^{V}-\mu P_{n}\\ S_{n}(0)=N_{n},\;P_{n}(0)=Z_{n} \end{array}\right..$$

Here, $N_{n}$ and $Z_{n}$ denote the host and pathogen density at the start of the epizootic in generation n, and μ is the pathogen decay rate, meaning the breakdown rate of infectious larval cadavers on the foliage [63]. This model arises via moment closure from the assumption that the host population exhibits heterogeneity in the pathogen’s infection rate, following a distribution with mean $\bar{\nu }$ and squared coefficient of variation^2^ V [57, supporting text]. Moderate values of V produce long-period, large-amplitude population cycles that are in qualitative agreement with observations of population fluctuations in many forest insects [15,19]. Because larvae by definition do not reproduce, this model describes the change in the host population that results from pathogen infection in the absence of host reproduction.

Multiple generations are coupled together by the following long-term discrete model:


(2.2) $$\left\{ \begin{array}{l} N_{n+1}=\lambda N_{n}(1-I_{n})\\ Z_{n+1}=\phi N_{n}I_{n}+\gamma Z_{n}\\ I_{n}=1-\frac{S_{n}(T)}{S_{n}(0)} \end{array}\right..$$

These equations describe host reproduction and pathogen overwintering. The symbol $I_{n}$ represents the fraction of larvae that die because of pathogen infection during the nth generation (recall that the pathogen is fatal). The density of surviving host larvae given by $S_{n}(T)=(1-I_{n})S_{n}(0)=(1-I_{n})N_{n}$, gives rise to adults that lay the next generation of eggs, at a fixed rate λ. Because we assume that λ allows for the non-disease mortality that occurs before and after the epizootic, we refer to λ as ‘net fecundity’. The discrete-generation model assumes that the eggs lay dormant throughout the winter and hatch in the spring. The $N_{n}I_{n}$ victims of the pathogen become infectious cadavers, which have net over-winter survival rate ϕ. Because ϕ allows for both pathogen over-wintering and the much higher susceptibility of hatching larvae, we allow ϕ>1. The parameter γ then allows for long-term pathogen survival in the environment.

### 2.2. Seasonal discrete-continuous hybrid models: the general case

Consistent with the ecological literature, with a specific focus on insect-pathogen systems, we define the seasonal hybrid model class to be composed of three sub-models: (i) an initialization function mapping the discrete variables $X_{n}$ at generation *n* to the continuous variables $Y_{n}(0)$ at time *t* = 0 of generation *n*, (ii) a continuous-time ODE model for $Y_{n}(t)$ parametrized by $X_{n}$, and (iii) a discrete-time dynamical system mapping $X_{n}$ to $X_{n+1}$, parametrized by the final-time continuous-time variables $Y_{n}(T)$. As we will show below, despite the coupled nature and complexity of (i)–(iii), our method is able to identify each of the sub-models (i)–(iii) from intermittent noisy measurements of the discrete and continuous variables.

More specifically, the model in (2.1) can be cast into this framework as follows. Letting $X_{n}=(N_{n},Z_{n})$ be the discrete-time model states described in (2.2) and $Y_{n}(t)=(S_{n}(t),P_{n}(t))$ be the continuous-time states described in (2.1), the hybrid model combining (2.1) and (2.2) has the following general form:


(2.3) $$\begin{array}{rl} X_{0} & \in R^{D}, \end{array}$$

(2.4) $$\begin{array}{rl} Y_{n}(0) & =h(X_{n}), \end{array}$$

(2.5) $$\begin{array}{rl} {\dot{Y}}_{n} & =g(Y_{n}(t),X_{n}), \end{array}$$

(2.6) $$\begin{array}{rl} X_{n+1} & =F(X_{n},Y_{n}(T)) \end{array}$$

for $X_{n}\in {\mathbb{R}}^{D},Y_{n}\in {\mathbb{R}}^{d}$ and some fixed T>0. This motivates our current focus, which is to identify F, g and h, for the general case (2.4)–(2.6), given measurement data and T. Note we have assumed that h depends only on the discrete (long-time) variable $X_{n}\in {\mathbb{R}}^{D}$, which is primarily for convenience, so that knowledge of $X_{0}$ is all that is needed to simulate the full system (2.4)–(2.6) given F, g and h. Our strategy will be to assume that F, g and h can all be represented as a linear combination of terms from a library 𝕃, and that 𝕃 is made sufficiently large and with sufficiently many terms that might be appropriate for the problem at hand.

While the equations in (2.3)–(2.6) capture a broad class of dynamics, we highlight some interesting extensions. It is possible to replace the dependence of F on $Y_{n}(T)$ with dependence on all of $Y_{n}(t),t\in [0,T]$. Further extensions of this model ((2.1) and (2.2)) include delayed pathogen interactions, genetic drift and stochasticity [17,19,64]. The method presented here is generally applicable to these extensions but may require including additional features in the library. This is certainly the case for the identification of stochastic hybrid models, and we aim to pursue that extension in future work.

We note that an alternative approach is to employ simplifying assumptions in order to replace the short-time model (2.5) with a fully discrete map, as undertaken in [19]. There, the fraction $I_{n}$ of infected hosts in (2.2) is approximated directly from $\left(N_{n},Z_{n}\right)$, removing the need to simulate the continuous time variables $\left(S_{n},P_{n}\right)$. While this is useful for studying long-term properties of the discrete model, the hybrid modelling framework is of more widespread relevance [23–27], and allows for much more flexible short-term dynamics. We defer discovery of purely discrete surrogate models for (2.3)–(2.6) to future work.

## 3. Methods

The main result of this article is that the discrete-continuous seasonal dynamics (2.3)–(2.6) can be identified from data with moderate measurement noise using the sparse weak-form equation learning approach proposed in this section. The proposed method is an extension and merger of two existing nonlinear dynamics identification algorithms, WSINDy [10,11] and WENDy [9]. The method is suitable for incomplete and irregularly collected data, with gaps in observations sometimes stretching over several years, as commonly seen in ecology given the inherent difficulties of collecting field data.

In what follows, we provide an overview of weak-form inference in §3.1, including the WSINDy and WENDy algorithms [9–11]. Specific advancements of these methods for the current setting of inference for hybrid discrete-continuous models of the form (2.4)–(2.6) is included in §3.2. We refer readers to appendix A, which contains several notation tables with mathematical symbols used throughout.

### 3.1. Weak-form equation learning and parameter inference

The equations (2.3)–(2.6) are known as the strong form of the differential equations, a form that is characterized by specifying that the equation holds pointwise in time, for all t. In contrast, the *weak form* of a differential equation specifies that the equation holds when integrated in time with respect to an arbitrary function referred to as the *test function*. The crux of weak-form methods relies on choosing the right test functions to achieve a given objective. For example, when the objective is inference from noisy data, smoothness of the test functions will be an important point. We discuss this in detail in the next subsections.

Weak-form inference methods have origins in parameter estimation dating back to the 1950s (e.g. [65]). In some disciplines, this is known as the modulating function technique [66]. Weak-form equation learning, which combines parameter estimation with model selection, is a more recent pursuit, and together with works published by authors of the current article [10,11,67–69], has seen many new developments in recent years [47,70–77].

Another recent pursuit in the context of parameter estimation is to successively improve weak-form estimates by iteratively incorporating the action of the test function basis Φ into the covariance structure for the weak-form residual. This is known as the WENDy method, or weak-form estimation of nonlinear dynamics, and has been shown to be advantageous over forward solver-based parameter estimation (such as nonlinear least squares) in a variety of ODE models of biological relevance [9].

In the current work, we combine the WENDy and WSINDy methods for the first time, arriving at an efficient inference framework for simultaneously selecting model terms and estimating parameters. Below, we review the WENDy and WSINDy methods.

**Strong-form model equation learning**. Given observations $𝐘=({𝐲}_{1},\ldots ,{𝐲}_{M})$ of a system’s state variables $y(t)\in {\mathbb{R}}^{d}$ where ${𝐲}_{i}$ are noisy observations of $y(t_{i})$ and $𝐭=(t_{0},\ldots ,t_{M})$ is a series of time points,^3^ the goal of *equation learning* is to identify an appropriate evolution equation


(3.1) $$y_{+}(t)=F(y(t)).$$

For discrete-time systems, the left-hand side is defined as $y_{+}(t)=y(t+\Delta )$ for a specified time interval Δ; for continuous-time systems $y_{+}(t)=\frac{\text{d}}{\text{d}t}y(t)$. Rather than merely approximating the map F from y to $y_{+}$, equation learning represents F as a human-readable function that can be used to infer the underlying mechanisms driving the system, and thus be used to further our understanding of the ecology of these systems, facilitate their control and inform future experimental designs.

The sparse identification of nonlinear dynamics (SINDy) equation learning algorithm [40] assumes that F admits a sparse expansion^4^


(3.2) $$\begin{array}{ccc} & F_{i}(y)={𝐖}_{1i}f_{1}(y)+\cdots +{𝐖}_{Ji}f_{J}(y),\;i=1,\ldots ,d & \end{array}$$

in terms of a *trial function library* $𝕃=(f_{j}{)}_{j=1}^{J}$, with each $f_{j}\in 𝕃$ having a meaningful interpretation relevant to the process being modelled. Here, $𝐖=({𝐖}_{ji}{)}_{j=1,i=1}^{J,d}$ is a *weight matrix* which is assumed to be sparse, with non-zero coefficient ${𝐖}_{ji}$ indicating that $f_{j}$ is an important mechanism in the dynamics of $y_{i}$. For instance, if y=(S,P) is a host–pathogen system, the term f(y)=−PS appearing in the $S_{+}$ equation would indicate that the host population decreases at a rate linear in the pathogen density, whereas the term $-\left(\frac{P}{1+P}\right)S$ would indicate that the rate of decrease levels off at high pathogen density, possibly capturing the effects of crowding or resource scarcity in the pathogen population.

The choice of an appropriate trial function library 𝕃 is highly dependent on the system under consideration. Automatic selection of 𝕃 is an ongoing research area [78,79]. Polynomial libraries are often an excellent initial choice due to their computational efficiency, interpretability, expressivity (i.e. all sufficiently smooth functions can be approximated by polynomials) and ubiquity in models of natural phenomena. On the other hand, low data quality may lead to multi-collinearity between library terms (including polynomial terms), preventing reliable selection of terms. In this case, expert knowledge about the system at hand is necessary to curate the trial function library [49]. Motivated by their frequent appearance in models of population dynamics, we employ polynomial libraries in this work, together with an automated library incrementation step as outlined in the following text.

**Weak-form model equation learning**. In *weak-form* learning, the requirement that the dynamics of y are governed by the so-called *strong-form* equation (3.1), holding at every t, is weakened as follows. First, the function t→F(y(t)) is assumed to belong to a space of functions X of square-integrable over a time window [0,T]. The space X is denoted as


$$L_{2}([0,T];{\mathbb{R}}^{d})=\{g:[0,T]\to {\mathbb{R}}^{d}|\int_{0}^{T}\|g(t){\|}_{2}^{2}dt<\infty \}$$

for continuous-time systems, and


$$\ell_{2}([M];{\mathbb{R}}^{d})=\{g:[M]\to {\mathbb{R}}^{d}|\sum_{n=0}^{M}\|g(n){\|}_{2}^{2}<\infty \}$$

for discrete-time systems, where [M]={0,1,2,…,M}, and M=⌊T/Δ⌋. We then let $X^{*}$ be the dual of X, that is, the space of continuous linear functionals φ:X→ℝ (e.g. [80, ch. 2] for an overview of function spaces and their duals). The *weak form* of (3.1) is then defined as


(3.3) $$\begin{array}{ccc} & \varphi (y_{+}(\cdot ))=\varphi (F(y(\cdot ))\;\text{for all}\varphi \in X^{*}. & \end{array}$$

When X is $L_{2}$ (and similarly for $\ell_{2}$), it holds that


(3.4) $$\begin{array}{ccc} & \varphi (g)=\int_{0}^{T}\phi (t)\cdot g(t)dt:=\left\langle \phi ,g\right\rangle & \end{array}$$

for all $\varphi \in X^{*}$, where ϕ∈X is some representer test function, and ⟨⋅,⋅⟩ is the $L_{2}$ inner product (Riesz’s Theorem [80, ch. 3]). We then assume that F admits the same representation as in (3.2).

The weak form (3.3) was originally developed to solve partial differential equations with jumps in the solution or other non-smooth characteristics [81] that do not satisfy the strong-form of the equation. This property makes the weak form beneficial when considering data corrupted by measurement noise, which can be interpreted as a solution to (3.3) holding on a suitable *subspace* $\Phi \subset X^{*}$. Inference methods that employ the weak form hinge on selecting an appropriate finite-dimensional subspace Φ, known as the *test function basis*, on which to enforce the weak form (3.3).

#### 3.1.1. WSINDy

We now briefly review the WSINDy algorithm (Weak-form SINDy [10,11]) and describe its application in continuous and discrete contexts. In both cases, a trial function library $𝕃=(f_{1},\ldots ,f_{J})$ and a test function basis $\Phi =(\phi_{1},\ldots ,\phi_{K})$ can be specified by the user. We now describe guidelines for choosing Φ, with specific test functions employed in this work included in appendix B.2.

**Continuous time**. When the data 𝐘 are assumed to come from a continuous-time process y(t) which obeys an ordinary differential equation, integration by parts can be leveraged in the weak form to avoid computation of numerical derivatives. For this, the test functions must at least be continuously differentiable; however, as identified in [10], requiring test functions to have higher order continuous derivatives can increase accuracy. In many cases though, the choice of Φ can be informed based on the data 𝐘 [11, appendix A].

**Discrete time**. In discrete time, there are no issues with discretizing integrals or numerically approximating derivatives. The test functions can simply be taken as δ-functions, $\phi_{k}=\delta_{k\Delta }$, defined by


$$\left\langle \delta_{k\Delta },y\right\rangle =y(k\Delta )$$

for a specified time point kΔ, with k∈ℕ and Δ the fixed time interval. This is the strategy employed in the current work, although nothing precludes us from employing $\phi_{k}$ with a greater support in time. We discuss this more in §5.

**Weak-form linear system**. For given 𝕃 and Φ, the *weak-form linear system* (𝐆,𝐁) is then formed by discretizing the weak-form equation (3.3) at the given data point values 𝐘. The entries of the matrix $𝐆\in {\mathbb{R}}^{K\times J}$ are given by


(3.5) $$G_{kj}=\langle \;\langle \phi_{k},f_{j}(Y)\rangle \;\rangle ,$$

where ⟨⟨⋅,⋅⟩⟩ is a discretized version of the inner product (3.4), taken for continuous systems to be the trapezoidal rule,


$$\begin{array}{rl} & \langle \;\langle \phi ,\;f(Y)\rangle \;\rangle =\\ & \;\sum_{i=0}^{M-1}\left(\frac{t_{i+1}-t_{i}}{2}\right)\left[\phi \;(t_{i})\;f(y_{i})+\phi \;(t_{i+1})\;f\;(y_{i+1})\right]. \end{array}$$

In discrete time systems, we simply have $\langle \;\langle \cdot ,\cdot \rangle \;\rangle ={\left\langle \cdot ,\cdot \right\rangle }_{\ell_{2}}$, the $\ell_{2}$ inner product.

In continuous time, the entries of 𝐁 are computed using integration by parts for (3.4), yielding


(3.6) $$B_{ki}=\phi_{k}Y_{i}{|}_{t=t_{0}}^{t=t_{M}}-\langle \;\langle \frac{d}{dt}\phi_{k},Y_{i}\rangle \;\rangle ,$$

where ${𝐘}_{i}$ is the time series of the observed ith coordinate of $y\in {\mathbb{R}}^{d}$. It is then assumed that $\phi_{k}(t_{0})=\phi_{k}(t_{M})=0$ to remove the boundary terms (first term on the right-hand side of (3.6)) from ${𝐁}_{ki}$.

In discrete time, we define ${𝐘}^{+}={𝐓}_{+}𝐘$, where ${𝐓}_{+}$ performs a shift forward in time, ${𝐘}_{mi}^{+}={𝐲}_{i}((m+1)\Delta )$, and similarly ${𝐓}_{-}$ performs a backwards shift, to get


(3.7) $$\begin{array}{rl} B_{ki} & =\langle \;\langle \phi_{k},Y_{i}^{+}\rangle \;\rangle =\langle \;\langle T_{-}\phi_{k},Y_{i}\rangle \;\rangle \;\;+\\ & [\phi_{k}(M\Delta )y_{i}((M+1)\Delta )-\phi_{k}(-\Delta )y_{i}(0)], \end{array}$$

where again we assume ϕ(−Δ)=ϕ(MΔ)=0 to remove the boundary terms. Placing operators in the 𝐁 matrix onto the test functions is central to the WENDy algorithm, outlined in the following text.

**Sparse regression**. Once (𝐆,𝐁) is formed, a sparse regression problem is solved for the set of weights as follows:


(3.8) $$\hat{W}={\text{argmin}}_{W}\sum_{i=1}^{d}\|GW_{i}-B_{i}{\|}_{2}^{2}+\kappa^{2}\|W_{i}{\|}_{0}.$$

In SINDy [40], the sequential thresholding least squares (STLS) algorithm [40,41] is employed to solve (3.8) which involves truncation of terms with coefficients $|{𝐖}_{ji}|<\kappa$, in other words uniform thresholding according to κ. In WSINDy, (3.8) is solved using the MSTLS algorithm (modified STLS), which uses nonuniform thresholding and performs a grid search to select the sparsity threshold κ. In MSTLS, thresholding intervals $I_{ij}^{\kappa }$ are defined by


(3.9) $$I_{ji}^{\kappa }=\left[\kappa \max\left\{1,\frac{\|B_{i}{\|}_{2}}{\|G_{j}{\|}_{2}}\right\},\;\kappa^{-1}\min\left\{1,\frac{\|B_{i}{\|}_{2}}{\|G_{j}{\|}_{2}}\right\}\right],$$

and at each step, $|{𝐖}_{ji}|\notin I_{ji}^{\kappa }$ is removed. For example, with $\kappa =10^{-2}$, this says that the overall term magnitude $\|{𝐆}_{j}{𝐖}_{ji}{\|}_{2}$ as well as the coefficient value $|{𝐖}_{ji}|$ are each restricted to lie within two orders of magnitude from $\|{𝐁}_{i}{\|}_{2}$ and 1, respectively.

#### 3.1.2. WENDy

The WENDy algorithm [9] is a forward-solver-free method for estimating parameters in nonlinear dynamical systems for which the model form is known. The only requirement is that the systems are linear in parameters (as in (3.2)), agreeing with the WSINDy construction just described. That is, the sparsity aspect of WSINDy is removed. A very simple approach to solving this problem would be to set κ=0 in (3.8), leading to an ordinary least-squares problem. From a statistical point of view, however, the underlying assumption of least squares is that the ith residual vector


$${𝐑}_{i}=𝐆{𝐖}_{i}-{𝐁}_{i}$$

has uncorrelated entries with zero mean. This would be a fundamentally incorrect assumption, however, because 𝐆 is in most cases nonlinear in the data 𝐘 and because the weak form introduces correlations in time. The idea behind WENDy is to utilize the correlations imparted by the weak form, and to correct for nonlinearities, in order to improve parameter estimates and quantify uncertainty.

In order to incorporate the inherent correlations in 𝐑 into an estimate for 𝐖, we first define the vectorized quantities 𝐲=𝗏𝖾𝖼(𝐘), 𝐰=𝗏𝖾𝖼(𝐖) and subsequently the vectorized residual $𝐫(𝐰;𝐲)=\text{𝗏𝖾𝖼}(𝐑)\in {\mathbb{R}}^{Kd}$, with entries


$$r_{(i-1)K+k}(w;y)=R_{ki}=\langle \;\langle D^{*}\phi_{k},Y_{i}\rangle \;\rangle -\langle \;\langle \phi_{k},L(Y)W_{i}\rangle \;\rangle .$$

Here, D denotes either $\frac{\text{d}}{\text{d}t}$ or the forward shift operator ${𝐓}^{+}$, depending on continuous or discrete systems, and $D^{*}$ is its adjoint ($-\frac{\text{d}}{\text{d}t}$ for continuous systems and ${𝐓}^{-}$ for discrete systems). If the samples are perturbed, $𝐲={𝐲}^{⋆}+\delta 𝐲$, we can propagate the effect of the errors δ𝐲 into the parameter estimates $\hat{𝐰}$ by Taylor expanding 𝐫 evaluated at the true weights ${𝐰}^{⋆}$ around the unperturbed data ${𝐲}^{⋆}$,


$$r(w^{⋆};y)\approx r(w^{⋆};y^{⋆})+L(w^{⋆})\delta y+O(\|\delta y{\|}^{2}),$$

where $𝐋({𝐰}^{⋆})$ is a linear operator defined as the Fréchet derivative


$$L(w^{⋆})\delta y=\lim_{\epsilon \to 0}\frac{1}{\epsilon }r(w^{⋆};y^{⋆}+\epsilon \delta y){|}_{\epsilon =0}.$$

Moreover, $𝐋({𝐰}^{⋆})$ is linear in ${𝐰}^{⋆}$, computed using


(3.10) $$L(w^{⋆}):=L^{\left(0\right)}(\Phi )+\sum_{\ell =1}^{Jd}w_{\ell }^{⋆}L^{\left(\ell \right)}(\Phi ,y),$$

where the Jd+1 matrices ${𝐋}^{\left(0\right)},\ldots ,{𝐋}^{\left(Jd\right)}$, referred to as the *covariance factors*, depend only on Φ, 𝕃 and 𝐲 and can thus can be pre-computed without knowledge of ${𝐰}^{⋆}$ (see [9] for details).

When δ𝐲 is normally distributed with mean zero and covariance Σ, then the residuals $𝐫({𝐰}^{⋆};𝐲)$ are approximately normal with mean zero and covariance


$$C(w^{⋆})=L(w^{⋆})\Sigma L(w^{⋆}{)}^{T},$$

thus an approximate set of weights $\hat{𝐰}\approx {𝐰}^{⋆}$ can be obtained by solving the generalized least squares (GLS) problem


(3.11) $$\min_{w}(G_{{\;}_{\text{\textbackslash{}tiny}⊗}}w-b{)}^{T}C^{-1}(w)(G_{{\;}_{\text{\textbackslash{}tiny}⊗}}w-b),$$

where 𝐛=𝗏𝖾𝖼(𝐁), ${𝐆}_{{,}_{⊗}}={𝐈}^{\left(d\right)}⊗𝐆$ and ${𝐈}^{\left(d\right)}$ is the identity in ${\mathbb{R}}^{d\times d}$. However, (3.11) introduces 𝐰 dependence into 𝐂. In WENDy this is dealt with using iterative reweighted least squares (IRLSs), starting from an initial ordinary least squares solve ${𝐰}^{\left(0\right)}=({𝐆}_{{,}_{⊗}}{)}^{†}𝐛$, before updating the covariance $𝐂({𝐰}^{\left(0\right)})$ and performing GLS for a new set of weights (see lines 7–12 of algorithm 2).

#### 3.1.3. WSINDy–WENDy

In this work, we combine the WSINDy and WENDy approaches. The former is employed to perform efficient model selection, while WENDy is used to estimate the selected model’s parameters more accurately and more precisely [9].

**MSTLS–WENDy**. The first step in this combination is the new sparse regression algorithm MSTLS-WENDy, provided in algorithm 2, whereby WENDy replaces the usual ordinary least squares steps in MSTLS. MSTLS-WENDy outputs an approximate set of weights $\hat{𝐰}$, an approximate residual covariance matrix $\hat{𝐂}=𝐂(\hat{𝐰})$, and an approximate parameter covariance matrix $\hat{𝐒}$.^5^ Hyperparameters for MSTLS-WENDy include WENDy hyperparameters τ and MaxIts, which represent the stopping tolerance and maximum number of WENDy iterations, respectively, as well as the candidate thresholds κ used in the MSTLS grid search.

**Library incrementation**. To increase efficiency, we sequentially increment the library 𝕃 using covariance information from WENDy to estimate the expected norm of the residual 𝐫. In the current setting, parametrized libraries can quickly grow to exceed computational resources. To circumvent this, we consider a nested sequence of libraries ${𝕃}^{\left(0\right)}\subset {𝕃}^{\left(1\right)}\subset \cdots$ together with an incrementation operator ${𝕃}_{+}$ defined by


(3.12) $$L_{+}(L^{\left(j\right)})=L^{\left(j+1\right)}.$$

We then define an incrementation criteria based on the outputs $({\hat{𝐰}}^{\left(\ell \right)}$, ${\hat{𝐂}}^{\left(\ell \right)},{\hat{𝐒}}^{\left(\ell \right)})$ and weak-form matrix ${𝐆}_{{,}_{⊗}}^{\left(\ell \right)}$ of MSTLS-WENDy with library ${𝕃}^{\left(\ell \right)}$ as follows. The WENDy residual ${\hat{𝐫}}^{\left(\ell \right)}={𝐆}_{{,}_{⊗}}^{\left(\ell \right)}{\hat{𝐰}}^{\left(\ell \right)}-𝐛$ is approximately normally distributed [9],


$${\hat{r}}^{\left(\ell \right)}∼N(\text{0},{\hat{C}}^{\left(\ell \right)}).$$

Similarly, we have that the mean squared residual is approximately normally distributed,^6^


$$r^{\left(\ell \right)}=\frac{1}{Kd}\sum_{k=1}^{Kd}({\hat{r}}_{k}^{\left(\ell \right)}{)}^{2}∼N({\hat{M}}^{\left(\ell \right)},{\hat{V}}^{\left(\ell \right)}),$$

where ${\hat{M}}^{\left(\ell \right)}$ and ${\hat{V}}^{\left(\ell \right)}$ are the sample mean and covariance of the diagonal of ${\hat{𝐂}}^{\left(\ell \right)}.$ A criterion to increment the library to ${𝕃}^{\left(\ell +1\right)}$, for a given confidence level c∈(0,1), is then


(3.13) $$\begin{array}{rl} & \text{LibIncFun}({\hat{w}}^{\left(\ell \right)},{\hat{C}}^{\left(\ell \right)},c)\\ & :=\{r^{\left(\ell \right)}>\max({\hat{M}}^{\left(\ell \right)}+\Psi^{-1}(c)\sqrt{{\hat{V}}^{\left(\ell \right)}},\;\text{tol}\|b{\|}_{\text{rms}}^{2})\}, \end{array}$$

where Ψ is the standard normal cumulative distribution function (CDF). A value of $\text{𝙻𝚒𝚋𝙸𝚗𝚌𝙵𝚞𝚗}({\hat{𝐰}}^{\left(\ell \right)},{\hat{𝐂}}^{\left(\ell \right)},c)=\text{𝚃𝚛𝚞𝚎}$, indicates that ${\hat{𝐫}}^{\left(\ell \right)}$ is outside of the confidence interval defined by c, which indicates that a better model fit might be warranted. In the event that the covariance is grossly underestimated, the method defaults to a simple residual-based criteria given by $r^{\left(\ell \right)}>\text{𝚝𝚘𝚕}\|𝐛{\|}_{\text{rms}}^{2}$, where 𝚝𝚘𝚕 =0.01 in the numerical experiments in the following text.

### 3.2. Weak-form sparse identification for seasonal discrete-continuous models

Our proposed approach to identify dynamical systems of the general seasonal hybrid form (2.4)–(2.6), which we will refer to as the WSINDy-Eco algorithm, is presented in algorithm 1. Given data of the form $𝐗=(X_{n},X_{n+1}{)}_{n\in I}$ and $𝐘=(Y_{n}({𝐭}_{n}){)}_{n\in I}$ for a finite set of generations I and time-grids $({𝐭}_{n}{)}_{n\in I}$, WSINDy-Eco outputs weight vectors $\left({\hat{𝐰}}^{\text{IC}},{\hat{𝐰}}^{Y},{\hat{𝐰}}^{X}\right)$ as well as libraries $\left({𝕃}^{\text{IC}},{𝕃}^{Y},{𝕃}^{X}\right)$ to define the learned model, estimates $\left({\hat{𝐂}}^{\text{IC}},{\hat{𝐂}}^{Y},{\hat{𝐂}}^{X}\right)$ for the covariances of the residuals for each sub-model, and parameter covariance estimates $\left({\hat{𝐒}}^{\text{IC}},{\hat{𝐒}}^{Y},{\hat{𝐒}}^{X}\right)$. Crucially, the parameter covariances allow for uncertainty quantification in a variety of predictive scenarios, as described in §§4.3 and 4.5. Choices of hyperparameters for WSINDy-Eco are presented in appendix C.2. Note that superscripts {IC,Y,X} refer to the initial conditions map (2.4), the continuous (Y) dynamics (2.5) and the discrete (X) dynamics (2.6), respectively (see appendix A for a full list of notations used).

A main component of WSINDy-Eco is the application of MSTLS-WENDy to parametrized libraries, dubbed MSTLS-WENDy-Par, described below in §3.2.1 with pseudocode given in algorithm 3 in the appendix. MSTLS-WENDy-Par utilizes recently developed methods for identifying parametric dependencies in PDEs [47]. However, our approach employs parametrized libraries to learn dependencies between the discrete and continuous variables in each of the sub-models (2.4)–(2.6).

In order to learn the discrete dynamics (2.6), an estimate of the continuous variables at time T, ${\hat{Y}}_{n}(T)$, is required, provided $Y_{n}(T)$ is not directly observed (which is often the case and is assumed here). In WSINDy-Eco, this is handled by simulating the learned continuous dynamics given by $\left({\hat{𝐰}}^{Y},{𝕃}^{Y}\right)$ using the learned initial conditions map given by $\left({\hat{𝐰}}^{\text{IC}},{𝕃}^{\text{IC}}\right)$, to obtain state estimates ${\hat{𝐘}}_{T}=({\hat{Y}}_{n}(T){)}_{n\in I}$. These estimates are then used in conjunction with the observed data $(Y_{n}({𝐭}_{n}){)}_{n\in I}$ to inform a covariance estimate ${\hat{\Sigma }}^{{\hat{𝐘}}_{T}}$ for use in the final MSTLS-WENDy-Par stage to approximate the discrete dynamics. This process is encompassed in the algorithm ForwardSim (algorithm 4). This is the only stage of WSINDy-Eco requiring forward solves (and is unique to the hybrid model framework); in particular, no forward solves are needed during model identification.

#### 3.2.1. Parametric MSTLS–WENDy

Each of the three sub-models (2.4)–(2.6) can be viewed as a parametrized equation, with state variables and parameters, of the form


(3.14) $$Du^{\left(s\right)}=f(u^{\left(s\right)};u^{\left(p\right)}).$$

In the initial conditions map (2.4), $u^{\left(s\right)}=Y_{n}(0)$, $u^{\left(p\right)}=X_{n}$, and D is simply the identity. For the continuous-time map (2.5), $u^{\left(s\right)}=Y_{n}(t)$, $u^{\left(p\right)}=X_{n}$ and $D=\frac{\text{d}}{\text{d}t}$. Finally, for the discrete-time map (2.6) $u^{\left(s\right)}=X_{n}$, $u^{\left(p\right)}=Y_{n}(T)$ and $D={𝐓}_{+}$. In [47], a sparse identification method is introduced to identify such parametric dependencies in dynamical systems, by building a tensorized library


$${𝕃}_{\left(s⊗p\right)}={𝕃}_{\left(s\right)}⊗{𝕃}_{\left(p\right)}$$

constructed from libraries ${𝕃}_{\left(s\right)}$ and ${𝕃}_{\left(p\right)}$ in the state and parametric variables, respectively. Applying MSTLS-WENDy to this setting is straightforward, assuming data ${𝐔}^{\left(s\right)},{𝐔}^{\left(p\right)}$ is available on $u^{\left(s\right)}$ and $u^{\left(p\right)}$, and data covariance matrices $\Sigma^{\left(s\right)},\Sigma^{\left(p\right)}$ are available such that approximately


$$\text{Cov}(\text{vec}(U^{\left(s\right)}))=\Sigma^{\left(s\right)},\;\text{Cov}(\text{vec}(U^{\left(p\right)}))=\Sigma^{\left(p\right)}.$$

The concatenated data $𝐮=(\text{𝗏𝖾𝖼}({𝐔}^{\left(s\right)}{)}^{T},\text{𝗏𝖾𝖼}({𝐔}^{\left(p\right)}{)}^{T}{)}^{T}$ then approximately satisfies


(3.15) $$\begin{array}{r} \Sigma^{\left(s⊗p\right)}:=\text{Cov}(u)=\left(\begin{array}{cc} \Sigma^{\left(s\right)} & 0\\ 0 & \Sigma^{\left(p\right)} \end{array}\right) \end{array}.$$

## 4. Numerical experiments

We now examine the performance of WSINDy-Eco applied to seasonal host–pathogen dynamics. To simplify exposition of the inference method, we restrict our examples to the epizootic models (2.1) and (2.2), which captures the key components of a seasonal hybrid model. An additional two-pathogen ecological example is included in appendix C, and guidelines for application to more general problems can be found in the code repository https://github.com/MathBioCU/wsindy_eco (e.g. a forced spiking neuron example utilizing the FitzHugh–Nagumo model).

Below we describe the data generation process, several performance metrics and methods of uncertainty quantification, before diving into results concerning long-term prediction (§4.4). We show that the method predicts accurately over many cycles of the continuous-time variables (despite requiring accurate updates and reinitializations in terms of the discrete variables at every generation n). We then describe a human-in-the-loop modelling framework that leverages the uncertainty information provided by the WENDy regression approach in §4.5. Finally, we evaluate the performance of the method under different sampling strategies in §4.6, revealing that peak sampling (data collection during outbreaks, as is customary in field studies), provides a clear advantage over random sampling in terms of predictive accuracy.

### 4.1. Data generation process

We perform simulations of the epizootic model (2.2) with parameters λ=5.5, ϕ=7, γ=0.3, V=0.5, $\bar{\nu }=1$ and μ=0.01, based on previous results from the literature and field studies [19,20,64], and initial conditions $\left(N_{0},Z_{0})=(0.00713,0.0183\right)$ leading to the true continuous dynamics


(4.1) $$\begin{array}{rl} & \left\{ \begin{array}{l} S_{n}(0)=(1.0)N_{n}\\ P_{n}(0)=(1.0)Z_{n} \end{array}\right. \end{array}$$

(4.2) $$\begin{array}{rl} & \left\{ \begin{array}{l} {\dot{S}}_{n}(t)=-(1.0)S_{n}(t)P_{n}(t){\left(\frac{S_{n}(t)}{N_{n}}\right)}^{V}\\ {\dot{P}}_{n}(t)=(1.0)S_{n}(t)P_{n}(t){\left(\frac{S_{n}(t)}{N_{n}}\right)}^{V}-(0.01)P_{n}(t) \end{array}\right. \end{array}$$

and the discrete update


(4.3) $$\begin{array}{rl} & \left\{ \begin{array}{l} N_{n+1}=(5.5)S_{n}(T)\\ Z_{n+1}=(7.0)N_{n}-(7.0)S_{n}(T)+(0.3)Z_{n}. \end{array}\right. \end{array}$$

**Figure 1.**
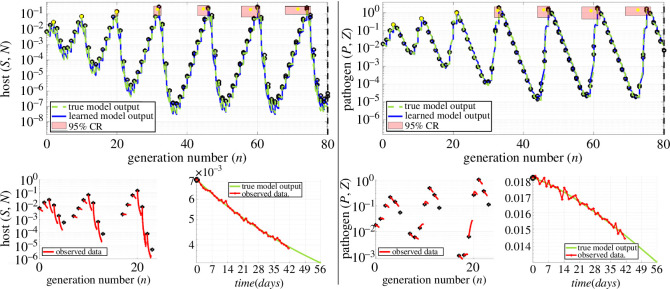
Example: 1% noise, accurate predictions up to 80 generations. Left and right columns visualize performance of WSINDy-ECO in predicting the host and pathogen densities, respectively. The top plots display learned model outputs overlapping the true model output for all 80 generations ($n_{\text{0.5}}(\hat{𝐰})=80$, black dashed lines), given |I|=18 samples from the first three host density peaks. The lower left plots in each column display the observed data, with black dots for discrete $\left(N_{n},Z_{n}\right)$ variables and red lines for continuous $\left(S_{n},P_{n}\right)$ variables over each generation. The modest 1% noise level in $\left(S_{n},P_{n}\right)$ is visualized for the n=0 generation in the bottom right plots of each column. By sampling the output parameters $\hat{𝐰}$ using the learned covariance $\hat{𝐒}$ and simulating the resulting models, 95% prediction regions can be ascribed to the peak locations and heights (red rectangles, with width and height given by 95% prediction intervals around mean peak location and height, respectively, with yellow dots for means), which in each case contain the true peaks and amplitudes. Note that although the parameter distribution is Gaussian $\left(𝐰∼N(\hat{𝐰},\hat{𝐒})\right)$, the uncertainty does not propagate linearly through the nonlinear dynamical system, resulting in non-symmetric prediction regions (hence yellow dots do not align with peaks).

**Figure 2.**
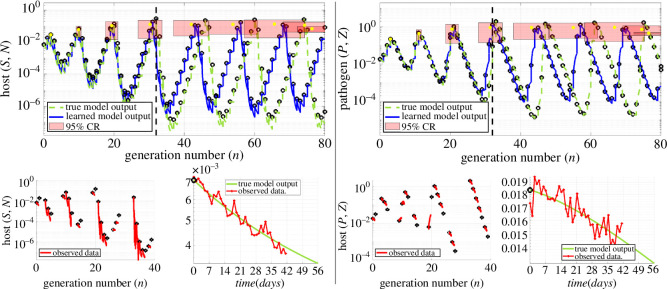
Example: 5% noise, accurate predictions up to 32 generations. Similar to figure 1, only now with 5% noise in (S,P) (see lower right plots in each column), and |I|=18 randomly sampled generations. The larger noise level limits accurate predictions to $n_{\text{0.5}}(\hat{𝐰})=32$ generations. The 95% prediction regions contain the true peaks up to $n_{\text{0.5}}(\hat{𝐰})$, after which the 95% CI for peak location still contains the true peak, but the true heights fall narrowly outside of the 95% CI.

Here, a duration of T=56 days is chosen to correspond to an eight-week epizootic period of the spongy moth [64]. See figures 1 and 2 for the host–pathogen dynamics that result from this model (dashed green lines in the top row plots).

The method introduced here requires data $𝐗=(X_{n},X_{n+1}{)}_{n\in I}$ on the discrete variables and data $𝐘=(Y_{n}({𝐭}_{n}){)}_{n\in I}$ on the continuous variables, over a finite set of generations I and time-grids $\{{𝐭}_{n}{\}}_{n\in I}$. To test our method, we simulate the model (4.1)–(4.3) for $n_{\max}=80$ generations, with I either sampled uniformly at random from the first 40 generations or clustered near peaks of the host density $N_{n}$. We fix the time-grids ${𝐭}_{n}=𝐭=(0,\Delta t,\ldots ,M\Delta t)$ across all generations and we use the realistic regime of one sample per day for 42 days (42 samples per generation), that is


(4.4) Δt=1,MΔt=(0.75)T=42.

We study the performance of the method while varying I and the noise level $\sigma_{NR}$ (defined below).

To emulate the effects of measurement noise, we apply log-normal noise to the continuous variables (S,P), and leave the discrete variables (N,Z) noise-free. This is reasonable because the continuous variables are typically population counts gathered in the field to represent the full population, while the discrete variables are aggregate quantities observed on a yearly basis. In addition, the presence of noise in both discrete and continuous variables would make the joint learning of states, parameters and the initial conditions difficult due to identifiability trade-offs (see §5 for more information). We use log-normal noise to preserve non-negativity of the population densities S and P. That is, for each n∈I, $t\in {𝐭}_{n}$ and Y∈{S,P}, we set


$$Y_{n}(t)=Y_{n}^{⋆}(t)\;\exp(\mu (t)+\sigma (t)\epsilon_{t})$$

for $\epsilon_{t}∼N(0,1)$ independent and identically distributed (i.i.d.), and μ(t) and σ(t) chosen such that $𝔼[Y_{n}(t)]=Y_{n}^{⋆}(t)$ and $𝕍[Y_{n}(t)]=\|Y_{n}^{⋆}({𝐭}_{n}){\|}_{\text{rms}}^{2}\sigma_{NR}^{2}$. Here, $Y_{n}^{⋆}(t)$ are the simulated (true, up to the numerical solver accuracy) solution values. We examine performance of the method under different *noise ratios* $\sigma_{NR}$, which approximately measures the relative error between the clean and noisy data, since


(4.5) $$\sigma_{NR}\approx \frac{\|Y_{n}^{⋆}(t_{n})-Y_{n}(t_{n}){\|}_{\text{rms}}}{\|Y_{n}^{⋆}(t_{n}){\|}_{\text{rms}}}.$$

Note that since $\|Y_{n}^{⋆}({𝐭}_{n}){\|}_{\text{rms}}^{2}$ varies with n, the data are heteroskedastic, with each generation having a different value of variance. However, this heteroskedasticity is naturally accounted for in the algorithm through the covariance estimation step outlined in appendix B.2.

### 4.2. Performance measures

We are primarily interested in the ability of the proposed method to identify the correct model and accurately predict the time series of population densities under different sampling and noise regimes. We focus on performance as a function of the number of generations observed |I| and the noise level $\sigma_{NR}$.


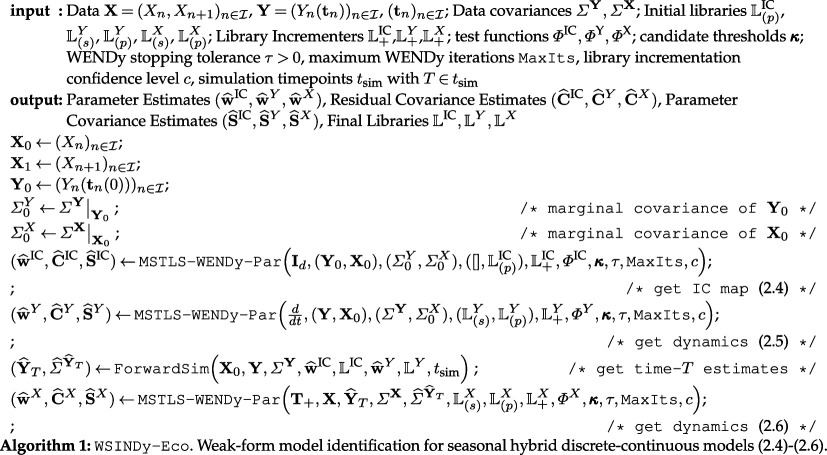


To measure the ability of the algorithm to correctly identify terms having non-zero coefficients in the true model (4.1)–(4.3), we use the *true positive ratio* (or the Jaccard Index) as in [82] defined by


(4.6) $$\text{TPR}(\hat{w})=\frac{\text{TP}}{\text{TP}+\text{FN}+\text{FP}},$$

where TP is the number of correctly identified non-zero coefficients, FN is the number of coefficients falsely identified as zero and FP is the number of coefficients falsely identified as non-zero. Identification of the correct model results in a TPR of 1, while identification of half of the correct terms and no incorrect additional terms results in TPR of 0.5. In this way, the TPR can be interpreted as the *fraction of correct terms*, which is more informative than simpler classification scores valued in {0,1} for {incorrect, correct} model identification. Here, we compute that TPR of each of the vectors $\left({\hat{𝐰}}^{\text{IC}},{\hat{𝐰}}^{Y},{\hat{𝐰}}^{X}\right)$ separately.

To assess the accuracy of the recovered coefficients, we measure the relative magnitude of the $\ell^{2}$ distance in parameter space using


(4.7) $$E_{2}(\hat{w}):=\frac{\|\hat{w}-w^{⋆}{\|}_{2}}{\|w^{⋆}{\|}_{2}},$$

where $\|\hat{𝐰}-{𝐰}^{⋆}{\|}_{2}$ corresponds to the sum of square differences between the estimated parameters and the true parameter values used to simulate the data, $\sum_{i}({\hat{w}}_{i}-w_{i}^{*}{)}^{2}$. This sum is then normalized by the total norm of the true parameter vector, $\sum_{i}(w_{i}^{*}{)}^{2}$ to provide the metric of relative parameter accuracy. Results for $E_{2}$ applied to ${\hat{𝐰}}^{X}$ are depicted in figure 7.

To measure prediction accuracy, we record the number of accurately predicted generations of the discrete variables, since this requires that all three sub-models (initial conditions map, continuous dynamics and discrete dynamics) have been accurately captured. Let $\Delta_{n}(\hat{𝐰})$ denote the relative cumulative error in the discrete variables up to generation n, given by


(4.8) $$\Delta_{n}(\hat{w}):=\sqrt{\frac{\sum_{n^{\prime }=0}^{n}{\left\|X_{n^{\prime }}^{⋆}-{\hat{X}}_{n^{\prime }}\right\|}_{2}^{2}}{\sum_{n^{\prime }=0}^{n}{\left\|X_{n^{\prime }}^{⋆}\right\|}_{2}^{2}}},$$

where $\hat{X}$ represents the learned model output with coefficients $\hat{𝐰}$ and $X^{⋆}$ the corresponding true model output, both initialized at the same discrete variables $X_{0}$. In words, $\Delta_{n}(\hat{𝐰})$ measures the goodness of fit between the learned trajectory and the noise-free true trajectory up to generation n, each simulated from the same initial conditions $X_{0}$, in the relative root-mean-squared sense. We report the number of generations that $\Delta_{n}$ remains below a certain tolerance,


(4.9) $$n_{\text{tol}}(\hat{w}):=\max\{n\leq n_{\max}\;:\;\Delta_{n}(\hat{w})\leq \text{tol}\}.$$

We set tol=0.5 throughout, which we empirically observe corresponds to the generation $n_{\text{tol}}$ at which the learned model visually departs from the ground truth. For example, figure 2 depicts an example dataset that achieved $n_{0.5}(\hat{𝐰})=32$, indicating that the learned model is able to forecast accurately (with a relative cumulative error not exceeding 50%) for 32 generations, given only $\left(N_{0},Z_{0}\right)$, the values of the discrete variables at generation n=0.

To generate statistics for the metrics (4.6), (4.7) and (4.9), for each noise level $\sigma_{NR}$ and number of observed generations |I|, the algorithm was run on R independently generated datasets, each with randomly generated noise. For peak sampling, the generation set I is fixed for each |I| and R=500, while for random sampling we sample I uniformly at random from {1,…,40}, and we use R=2000. These values of R were sufficient to yield stable statistics, with larger R needed for random sampling due to the fact that both the noise and the generation set I are random. Batch computations were performed on the University of Colorado Boulder’s Blanca Condo Cluster, while a Lenovo ThinkPad P14 laptop with AMD Ryzen Pro 7 processors and 48 GB of RAM was used for exemplary runs and associated walltimes.

### 4.3. Uncertainty quantification

WSINDy-Eco outputs a distributional model with asymptotically multivariate normal parameters


$$w^{\text{IC}}\;∼N({\hat{w}}^{\text{IC}},{\hat{S}}^{\text{IC}}),\;w^{Y}\;∼N({\hat{w}}^{Y},{\hat{S}}^{Y}),\;w^{X}\;∼N({\hat{w}}^{X},{\hat{S}}^{X}).$$

Collectively, we will refer to the full model output as $𝐰∼N(\hat{𝐰},\hat{𝐒})$. The simplest uncertainty metrics we can readily quantify are the confidence intervals around each parameter, obtained from $\text{𝖽𝗂𝖺𝗀}(\hat{𝐒})$. We will, however, also estimate the uncertainty in predictions using parametric bootstrap sampling: in §4.4, we quantify uncertainty in population peak times and amplitudes, and in §4.5, we present a human-in-the-loop modelling approach using various uncertainty quantification (UQ) metrics. In each case, we make 200 independent parameter draws ${𝐰}^{\left(\ell \right)}∼N(\hat{𝐰},\hat{𝐒})$, ℓ=1,…,200. Then, for each draw, the resulting model is constructed using the output libraries ${𝕃}^{\text{IC}},{𝕃}^{Y},{𝕃}^{X}$ and simulated from the same observed initial conditions. At each generation n=0,…,80, we compute histograms for the discrete host densities $\{N_{n}^{\left(\ell \right)}{\}}_{\ell =1}^{200}$ to visualize the growth of uncertainty over time (figures 4–6). Similarly, peaks $P_{1}^{\left(\ell \right)},P_{2}^{\left(\ell \right)},\ldots$ are computed for each draw ℓ=1,…,200, and prediction regions for each peak location and magnitude are found based on marginal sample order statistics, from 2.5th quantile to 97.5th quantile (figures 1 and 2).

### 4.4. Long-term prediction with uncertainty quantification

Figures 1 and 2 display typical results for 1% and 5% noise, respectively ($\sigma_{NR}=0.01,0.05$). In the 1% noise case, each of the sub-models (4.1)–(4.3) is identified correctly $\left(\text{TPR}({\hat{𝐰}}^{\text{IC}})=\text{TPR}({\hat{𝐰}}^{Y})=\text{TPR}({\hat{𝐰}}^{X})=1\right)$, with coefficient relative errors $E_{2}({\hat{𝐰}}^{\text{IC}})=7.8\times 10^{-4}$, $E_{2}({\hat{𝐰}}^{Y})=1.9\times 10^{-3}$ and $E_{2}({\hat{𝐰}}^{X})=1.9\times 10^{-5}$. The algorithm runs in 12 s on a modern laptop and allows for accurate prediction up to 80 generations (the maximum simulated). By sampling model parameters using the parameter covariance $\hat{𝐒}$, as described in §4.3, we can compute 95% prediction regions for the host and pathogen population peak locations and amplitudes, which in figure 1 are seen to contain the true peaks for all predicted cycles. For example, the fifth peak is estimated to occur approximately 44 years in the future with a 1-year margin.

The effects of larger measurement noise are depicted in figure 2, showing exemplary results for 5% noise in the $\left(S_{n},P_{n}\right)$ variables (see the bottom right subplots of each column for noise visualizations). The algorithm in this case is still able to correctly identify all sub-models (4.1)–(4.3), yet the coefficient errors $E_{2}({\hat{𝐰}}^{\text{IC}})=1.9\times 10^{-2}$, $E_{2}({\hat{𝐰}}^{Y})=9.4\times 10^{-3}$ and $E_{2}({\hat{𝐰}}^{X})=1.1\times 10^{-2}$ are now large enough to restrict the long-term predictive abilities of the learned model. Still, the learned model is able to capture the underlying true model output for 32 generations, after which the behaviour is qualitatively correct, namely a stable cycle appears to have been reached. The peak location and amplitude prediction regions in this case communicate that later population booms (peaks 5 and later) cannot be accurately predicted; however, we anticipate that in many cases only information on the next one–three peaks in is expected.

**Figure 3.**
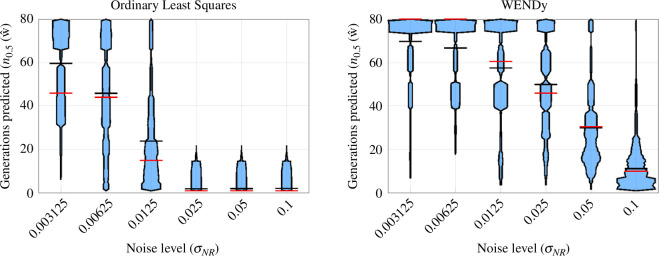
Advantages of WENDy regression in long-term prediction. Both plots depict the performance of WSINDy-Eco with data sampled from the first five host peaks (see figure 7 for sampling related performance), with ordinary least squares (OLS) regression on the left (MaxIts = 0) and WENDy regression on the right (MaxIts = 5). Employing WENDy increases predictive capabilities from 20 to 60 generations on average for data with 1.25% noise in the continuous variables. OLS does not allow for effective model learning at higher noise levels, leading to models that cannot predict well forward in time, while WENDy is still able to predict 45, 32 and 10 generations forward at 2.5%, 5% and 10% noise, respectively.

Finally, we report the overall advantages of the WENDy regression approach in affording long-term prediction in figure 3. Results are shown for sampling data from the first five host population peaks (see §4.6 for an in-depth treatment of sampling strategies). Simply using ordinary least squares (OLS, left plot), obtained by setting MaxIts = 0 in WSINDy-Eco, results in a lack of robust predictions when noise in the $\left(S_{n},P_{n}\right)$ variables exceeds 1.25%. On the other hand, accurately accounting for the errors-in-variables nature of the problem using WENDy with just five iterations (MaxIts = 5) enables accurate prediction for at least 10 generations on average for noise levels up to 10%, with 5% noise resulting in accurate predictions up to 32 generations, and 2.5% noise yielding accurate prediction up to 45 generations.

**Figure 4.**
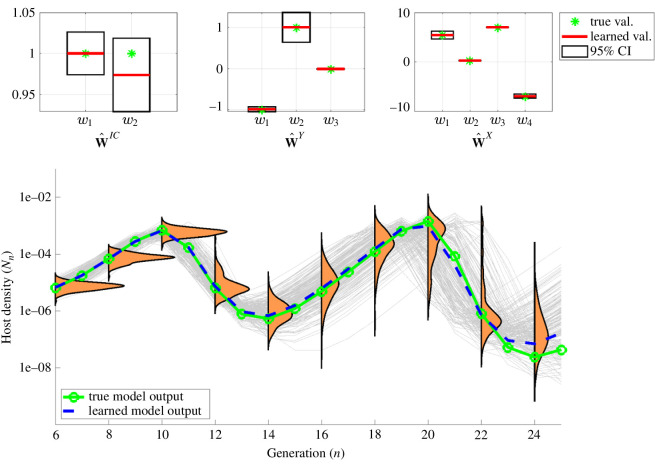
UQ under correct model identification. In this example with 5% noise (same data as in figure 2), the learned model is able to capture the data well (blue versus green curve). The added uncertainty quantification allows the WSINDy-Eco approach to provide a view into the validity and usefullness of the model. Confidence intervals around learned parameter values (top plots) each contain the true value and show model parameter uncertainty, indicating low uncertainty (narrow intervals) and good identification. Similarly, model trajectories (grey curves) obtained via parametric bootstrap samples from the WENDy parameter distribution, provide visualization of the uncertainty in state trajectories themselves. They also allow quantification of the spread of predictive values and propagation of uncertainty into forward predictions (orange kernel density estimates) at any point in time. Figure 5 displays corresponding results for the case of a misidentified model.

An additional way to quantify uncertainty is to understand how parameter uncertainty propagates into dynamical system trajectories. To see this, we sample model parameter sets (using parametric bootstrap as explained above), and plot the solution trajectories for each parameter set in figure 4. This allows us to assess prediction uncertainty in the state trajectories themselves, which are nonlinear transformations of the parameters (and parameter uncertainty). Histograms of solution samples at each generation n (orange kernel density estimates superimposed on the cloud of trajectories) reveal the spread of state values in the host population at each particular time slice. In this case, with the true model having been identified by the algorithm, the sampled state trajectories remain relatively tight and close to the true model trajectory. It is worth noting, however, that the state trajectories start to show reduced cohesiveness and heavier tails as the uncertainty increases the further into the future we are trying to predict.

**Figure 5.**
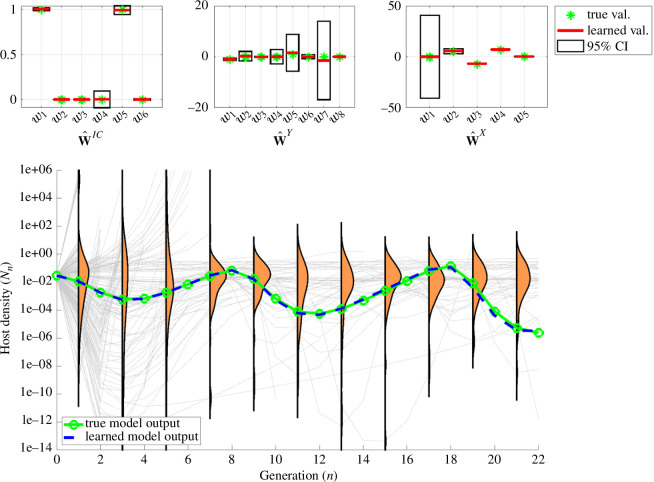
UQ under misidentified model, pre-manual model pruning. In the case of a misidentified model (also from data with 5% noise), the learned model output still fits the data well (blue versus green curves). However, uncertainty in trajectories is large, with different trajectories showing wildly different behaviour. Information provided by WENDy, combined with domain knowledge, can be used to manually rule out terms that do not seem biologically realistic; the next figure displays the results obtained from re-solving the system after eliminating $\left\{w_{2},w_{3},w_{4},w_{6}\right\}$ from ${\hat{𝐰}}^{\text{IC}}$, $\left\{w_{4},w_{6},w_{7}\right\}$ from ${\hat{𝐰}}^{Y}$ and $w_{1}$ from ${\hat{𝐰}}^{X}$, due to disproportionately smaller magnitudes and/or larger confidence intervals (top plots).

### 4.5. Human-in-the-loop modelling

A desirable quality of equation learning combined with covariance information (as afforded by the current method) is that models can be vetted by experts, through direct examination of the learned model terms, their respective coefficients and their corresponding covariance structure and confidence intervals. For example, figure 4 displays confidence intervals for the model described in figure 2 and shows that in this case each confidence interval contains the true value of the parameter (green star). Note that, normally we would expect 95% of our confidence intervals to contain the true values over repeated sampling. When confidence intervals are large, however, and include 0, this allows for a discussion about whether those terms should be excluded from the final model, as long as multiple testing strategies are employed.

In practice, however, there is no ground truth model, and it is quite possible that the learned model could be improved upon by further inspection from a researcher with expertise in the ecological systems being studied. It is also possible that low data quality and high noise can lead to suboptimal sparse recovery (see §4.6). In particular, if the model goodness of fit is not diminished in any meaningful way with the removal of terms, then by Occam’s razor the simpler model should be used. In the current framework, this can be tested immediately. For example, figure 5 displays the parameter confidence intervals (top) and forward simulation uncertainty (bottom) corresponding to the misidentified model in equation (4.10), with spurious terms noted in red (see (4.1)−(4.3)). The coefficient values and their confidence intervals are listed in table 1. At first glance, the terms look reasonable: the initial conditions map is linear, the continuous dynamics show competitive interactions (all terms with $S_{n}P_{n}$) with decay in the pathogen density $P_{n}$, while the discrete dynamics replace the host update $N_{n+1}$ with a linear combination of discrete and continuous host variables.

**Table 1.** Comparison of true and learned model coefficients, together with 95% confidence intervals, for the misidentified model in equation (4.10) (visualized in figure 5) and the corrected model obtained from eliminating $\left\{w_{2},w_{3},w_{4},w_{6}\right\}$ from ${\hat{𝐰}}^{\text{IC}}$, $\left\{w_{4},w_{6},w_{7}\right\}$ from ${\hat{𝐰}}^{Y}$, and $w_{1}$ from ${\hat{𝐰}}^{X}$ (visualized in figure 6).

coefficient	$w_{1}^{IC}$	$w_{2}^{IC}$	$w_{3}^{IC}$	$w_{4}^{IC}$	$w_{5}^{IC}$	$w_{6}^{IC}$
term	$N_{n}$	$Z_{n}$	1	$Z_{n}$	$N_{n}$	1
true model (equations (4.1)–(4.3))	1	0	0	1	0	0
misidentified model (figure 5)	1.00	−4.41 × 10^−5^	−2.10 × 10^−7^	0.99	1.17 × 10^−3^	−6.77× 10^−5^
(95% CI)	±0.017	±5.3 × 10^−4^	±6.2 × 10^−4^	±0.092	±0.049	±0.01
corrected model (figure 6)	1.00	0	0	0.99	0	0
(95% CI)	±0.015	0	0	±0.042	0	0

coefficient	$w_{1}^{Y}$	$w_{2}^{Y}$	$w_{3}^{Y}$	$w_{4}^{Y}$	$w_{5}^{Y}$	$w_{6}^{Y}$	$w_{7}^{Y}$	$w_{8}^{Y}$
term	$\frac{S_{n}^{1+V}}{N_{n}^{V}}P_{n}$	$S_{n}^{1+V}P_{n}$	$S_{n}P_{n}$	$\frac{S_{n}^{2}}{N_{n}^{V}}P_{n}$	$\frac{S_{n}^{1+V}}{N_{n}^{V}}P_{n}$	$\frac{S_{n}}{N_{n}^{V}}P_{n}$	$\frac{S_{n}^{2}}{N_{n}^{V}}P_{n}$	$P_{n}$
true model (equations (4.1)–(4.3))	−1	0	0	0	1	0	0	−0.01
misidentified model (figure 5)	0.99	0.22	−0.041	−0.01	1.53	−0.047	−1.49	−9.7 × 10^−3^
(95% CI)	±0.48	±1.8	±0.18	±2.9	±7.2	±0.8	±15	±1.8 × 10^−3^
corrected model (figure 6)	−1.02	0.36	−0.12	0	0.98	0	0	−0.01
(95% CI)	±0.13	±0.62	±0.68	0	±0.19	0	0	±1.1 × 10^−3^

coefficient	$w_{1}^{X}$	$w_{2}^{X}$	$w_{3}^{X}$	$w_{4}^{X}$	$w_{5}^{X}$
term	$\frac{S_{n}^{1+V}}{N_{n}^{V}}P_{n}$	$S_{n}^{1+V}P_{n}$	$S_{n}P_{n}$	$\frac{S_{n}^{2}}{N_{n}^{V}}P_{n}$	$\frac{S_{n}^{1+V}}{N_{n}^{V}}P_{n}$
true model (equations (4.1)–(4.3))	0	5.5	−7	7	0.3
misidentified model (figure 5)	-	5.42	−6.98	7.00	0.3
(95% CI)	1.1 × 10^−3^ ± 41	±2.4	±0.3	±0.037	±0.11
corrected model (figure 6)	0	5.45	−6.93	7.00	0.3
(95% CI)	0	±0.42	±0.23	±0.024	±0.088

**Figure 6.**
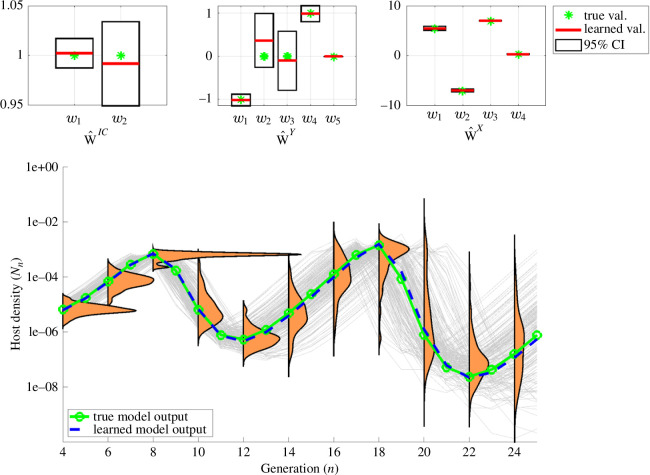
UQ for the misidentified model described in figure 5, post model pruning. After having eliminated $\left\{w_{2},w_{3},w_{4},w_{6}\right\}$ from ${\hat{𝐰}}^{\text{IC}}$, $\left\{w_{4},w_{6},w_{7}\right\}$ from ${\hat{𝐰}}^{Y}$ and $w_{1}$ from ${\hat{𝐰}}^{X}$ (see figure 5) and resolving the system using WENDy, a clearer trajectory pattern is revealed with tighter spread of oscillations. In particular, the next host population peak is predicted to occur at n=8 with a high degree of certainty.


(4.10) $$\left\{ \begin{array}{l} S_{n}(0)=w_{1}^{IC}N_{n}\\ +w_{2}^{IC}Z_{n}+w_{3}^{IC}\\ P_{n}(0)=w_{4}^{IC}Z_{n}\\ +w_{5}^{IC}N_{n}+w_{6}^{IC} \end{array}\right.\;\left\{ \begin{array}{l} {\dot{S}}_{n}(t)=S_{n}(t)P_{n}(t)[w_{1}^{Y}{\left(\frac{S_{n}(t)}{N_{n}}\right)}^{V}\\ \;+w_{2}^{Y}(S_{n}(t){)}^{V}+w_{3}^{Y}(1)+w_{4}^{Y}S_{n}(t)(N_{n}{)}^{-V}]\\ {\dot{P}}_{n}(t)=S_{n}(t)P_{n}(t)[w_{5}^{Y}{\left(\frac{S_{n}(t)}{N_{n}}\right)}^{V}+w_{6}^{Y}(N_{n}{)}^{-V}\\ \;+w_{7}^{Y}S_{n}(t)(N_{n}{)}^{-V}]+w_{8}^{Y}P_{n}(t) \end{array}\right.\;\left\{ \begin{array}{l} N_{n+1}=w_{1}^{X}N_{n}+w_{2}^{X}S_{n}(T)\\ Z_{n+1}=w_{3}^{X}S_{n}(T)+w_{4}^{X}N_{n}\\ +w_{5}^{X}Z_{n}. \end{array}\right.$$

To investigate the validity of this model, in the light of uncertainty in the dynamics the model describes, we simulate an ensemble of solution trajectories corresponding to a set of sampled parameters (sampled using parametric bootstrap as described earlier), as shown in figure 5 (bottom). Qualitatively, we see that a significant portion of trajectories diverge or stabilize early on. While such behaviour may be biologically realistic and valid (for instance host population blow-up could correspond to pathogen extinction), the observed oscillations provide some scrutiny of those mechanisms. Investigation of the coefficients $\hat{𝐰}$ and their respective confidence intervals (table 1) provides support for model modification by removal of terms. Confidence intervals around $w_{2},w_{3},w_{4},w_{6}$ of ${\hat{𝐰}}^{IC}$, $w_{4},w_{6},w_{7}$ of ${\hat{𝐰}}^{Y}$ and $w_{1}$ of ${\hat{𝐰}}^{X}$ indicate that the sign of the respective coefficients cannot be reasonably inferred, and thus a value of zero cannot be ruled out. In addition, the magnitudes of $w_{2},w_{3},w_{6}$ in ${\hat{𝐰}}^{IC}$ are of several orders smaller than the remaining coefficients in ${\hat{𝐰}}^{IC}$, which further advocates that their mechanisms might be insignificant in the seasonal reinitialization of host and pathogen densities.

We can therefore ‘manually’ set each of these coefficients to zero, and re-solve the differential equation system with the remaining terms using just the WENDy algorithm. The result is depicted in figure 6. Although this system still contains two spurious model terms, the ensemble of trajectories (bottom row) now better communicates the spread of oscillations in trajectories. For example, one can infer from the sharply peaked density that the next population boom will probably occur at generation n=8 with high confidence. Finally, to decide if we can formally adopt the pruned (reduced) model as the replacement for the original (full) model in equation (4.10), we would need to formally compare the full and reduced models using a model selection criterion such as for example AIC [6,83]. In our case, the reduced model does indeed achieve a lower AIC score of 771.14 (using equations (C 1) and (C 2) compared with the full model’s AIC score of 782.59, and thus the reduced model should be selected.

Overall, this process reveals a procedure by which expert knowledge can be used to modify equations and quickly assess hypotheses using the modified equations, as well as assess the uncertainty in the learned model and its predictions. We reiterate that the entire learning algorithm, including assessment and modification of the learned model as just described, occurs with no more than several minutes of computation time on a modern laptop.

### 4.6. Sampling strategies

Data collection (sampling) strategies are an important consideration in ecological applications, due in part to limited resources and challenges associated with field work. Specific questions may include when to sample, how many samples to collect, and during which years to collect samples. Several recent studies have sought to quantify the effect of sampling strategies on the performance of SINDy-type methods, concluding in some cases that sampling near fast dynamics is more informative than near slow dynamics [10,48], and that interpolation onto finer grids may be employed to overcome sparse measurements [49]. In this section we focus on realistic sampling strategies from the perspective of ecological field work. Namely, we discuss quantitative performance results from two different collection strategies, random sampling and peak sampling. The random sampling strategy involves sampling the generation set I uniformly at random from the first 40 generations, with |I|∈{12,16,20}. Peak sampling, which is the strategy typically employed in studies of outbreaking organisms, refers to sampling generations around host population peaks. Letting $n_{i}$ denote the location of the ith host population peak, we include $\{n_{i}-2,n_{i}-1,n_{i},n_{i}+1\}\subset I$ for each of the sampled peaks. We consider peak sampling from the first three, four or five peaks, and perform a direct comparison with the same corresponding randomly sampled number of generations |I|.

**Figure 7.**
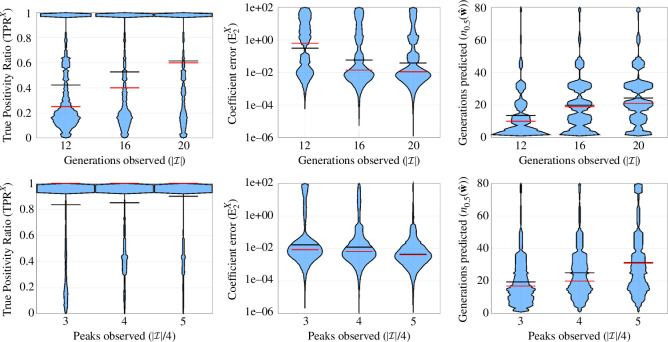
Violin plots comparing aggregate performance for random and peak sampling strategies. Left to right column: TPR${,}^{X}$ (model recovery accuracy for the discrete dynamics), $E_{2}^{X}$ (coefficient accuracy for discrete dynamics) and $n_{0.5}$ (prediction length) for data with 5% noise in the continuous variables equations (4.6)–(4.9). Top and bottom rows display results for random and peak sampling, respectively. In each violin plot, the sample mean and median are displayed with black and red lines, respectively. For visualization, values with $E_{2}^{X}>100$ have been removed, representing approximately 40%, 36% and 19% of values for random sampling and 6.2%, 1.8% and 2.6% of values for peak sampling. It can be observed from the left plots that sampling three peaks only is sufficient to identify the dynamics in the vast majority of cases with average TPR above 0.8, whereas random sampling average TPR is below 0.6 even with the equivalent of five peaks worth of data. Correspondingly, the right plots show that peak sampling with only three peaks doubles the accurate prediction length to 20 generations on average, compared with the equivalent data quantity random sampling with 12 generations.

Figure 7 top row presents violin plots summarizing the method performance when using random sampling, as data quantity (the number of generations sampled) increases, over 2000 simulated datasets for each |I|. Similarly, the bottom row of figure 7 presents violin plots summarizing the method performance when using peak sampling, as data quantity (the number of peaks sampled, with each peak having four generations) increases, over 500 simulated datasets for each number of peaks (|I|/4). The first column of the figure shows TPR (defined in equation (4.6)), with higher values conveying better performance at learning the correct model. The middle column of figure 7 presents coefficient relative error (defined in equation (4.7)), with higher values corresponding to worse performance. Finally, the right column of figure 7 presents the number of generations accurately predicted (metric defined in equation (4.9)), with higher values corresponding to greater number of accurately predicted generations and better method performance.

In the low peak-sampling regime, when only three peaks are observed, the learned models on average still robustly identify the discrete map (${\text{TPR}}^{X}\approx 1$, bottom left plot), compared with random sampling, which yields ${\text{TPR}}^{X}\approx 0.3$ on average (top left). Recall that correct identification of the discrete map relies on accurate identification of both the initial conditions and the continuous map (see algorithm 1), making TPR${,}^{X}$ a good indicator of accurate identification of the full model. This directly correlates with the observed prediction accuracy, as measured by $n_{0.5}(\hat{𝐰})$ (right plots), whereby three peaks are sufficient to predict up to 20 generations (with a long tail extending up to 80 generations), nearly twice that of random sampling. This is also reflected in the coefficient accuracy (middle plots), from which an improvement by two orders of magnitude (on average) is observed using peak sampling.

When five peaks are observed, results improve slightly, in particular $n_{0.5}(\hat{𝐰})$ on average increases to approximately 30 for peak sampling and approximately 20 for random sampling. However, random sampling still trails behind peak sampling with TPR${,}^{X}\approx 0.6$ compared with TPR${,}^{X}\approx 1$ for peak sampling, along with larger coefficient errors $E_{2}^{X}$. Additional performance metrics for the initial conditions and continuous dynamics are shown in figure 14 of the appendix, showing similar trends.

In summary, the bottleneck in predictive accuracy of the learned model is found to be identification of the correct model terms (e.g. as measured by the TPR). Peak sampling provides a clear advantage by offering sufficient data quality for the sparse regression routines to be effective even when few total generations are observed, indicating that host peak samples are more informative for long-term prediction. We conjecture that random sampling is a less effective strategy due to the higher probability of sampling in the slow, out-of-peak phase of the dynamics. Because host peaks rise and fall in relatively few generations, uniformly-at-random generation sampling is less likely to sample during the (highly dynamic) host peaks. This at least agrees with previous studies in which samples during fast dynamics are found to produce better results than samples during slow dynamics [10,48]. Overall, however, the proposed approach is able to work effectively with intermittent generation samples and data gaps, especially when peak sampling is employed, despite the log-normal noise model falling outside of the normality assumptions inherent to regression via WENDy thanks to the consistency properties of the embedded GLS routine which depend on only the first two moments [84–86].

## 5. Discussion

### 5.1. Summary

We present a general framework for rapidly learning hybrid discrete-continuous dynamics driven by strong seasonality with relevance to ecological systems. The resulting WSINDy-Eco algorithm runs in seconds on a modern laptop. Sparse model selection identifies dominant mechanisms in the dynamics which can easily be interpreted by the explicit model expressions, including complex coupling between the discrete and continuous variables. The method is well suited for a variety of sampling designs, including data with irregular sampling characterized by missing data for several generations. Distributional information about the WENDy parameter estimators afforded by the methodology [9] enables uncertainty quantification which can be used to provide confidence around model predictions, and to easily modify learned models, as described in §4.5. Moreover, the framework is not restricted to models in ecology, but can easily be applied to general hybrid systems featuring coupled discrete and continuous variables.

### 5.2. Extensions

A major benefit of the general weak formulation and library-based model learning is that libraries and test functions can also be tailored to suit different problems. Even though the library employed in this work is large, the terms in it were realistic for the problem at hand. For example, when we consider genetic drift and stochasticity in future work, to complement previous ecological studies [17,19,64], we will append additional terms to the library. We note also that our method need not be restricted to ecological settings and is directly applicable to seasonal hybrid systems, a ubiquitous modelling framework across the life sciences [12,54,87]. In our code repository (https://github.com/MathBioCU/wsindy_eco), we include resources for applications to other disciplines, including an additional example of a forced spiking neuron. In addition, different test functions for discrete-time inference may be used, reflecting different correlation structures if so desired.

### 5.3. Data quality and sparse regression

The data we considered here is meant to match realistic field studies, often limited to sampling a few times per week, often with 10–20 generations of data on hand [88–91]. Results did not vary significantly when the number of intergenerational samples M and sampling rate Δt were varied outside of the fixed values (4.4), with ΔtM∈(0.5T,T) and M≥24. Furthermore, we note that for the system (4.1)–(4.3), the coarsest sampling needed to identify the true model under zero-noise conditions is M=8 samples per generation, corresponding to roughly one sample per week, over nine (not necessarily consecutive) generations, which leads to all parameters estimated to within 0.08% relative error.

As in general, we will not know *a priori* what the noise distribution is, we have presented a general framework that we tested on a misspecified likelihood by treating log-normal noise using a GLS estimator within WENDy. Instead of tailoring to a specific likelihood (e.g. log-normal), which would require additional knowledge, we have relied on consistency properties of GLS, which depend only on correct specification of the first two moments, which our algorithm was able to handle well.

**Figure 8.**
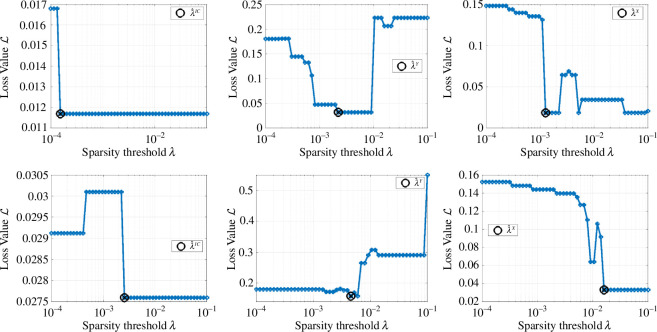
Visualization of sparsity threshold selection in MSTLS. Left to right: selection of thresholds for the initial conditions map, continuous dynamics and discrete dynamics. Top and bottom: 1% noise and 5% noise. There is a clear relationship between the selected threshold and the level of noise in the data, with higher noise levels corresponding to the need for (and the selection of) a larger threshold $\hat{\kappa }$, and consequently less complex models. In the case of the continuous dynamics (middle plots) the loss function becomes flatter, indicating that there are potentially multiple models describing the data equally well. In these examples all sub-models were identified correctly with the exception of the continuous dynamics at 5% noise, yet the recovered model is accurate enough to enable identification of the discrete dynamics (recall this depends on the outputs of the simulated learned continuous dynamics).

**Figure 14.**
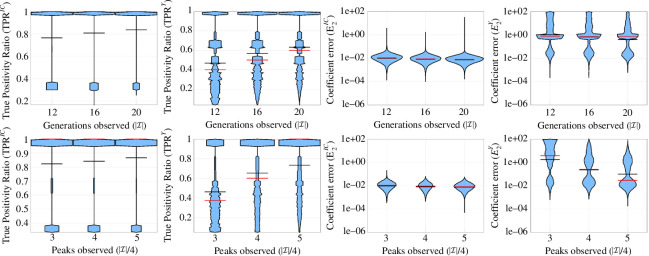
Violin plots comparing aggregate performance for random and peak sampling strategies (figure 7 cont.). Left to right: TPR${,}^{IC}$ (model recovery accuracy for the initial conditions map), TPR${,}^{Y}$ (model recovery accuracy for the continuous dynamics), $E_{2}^{IC}$ (coefficient accuracy for initial conditions map), $E_{2}^{Y}$ (coefficient accuracy for the continuous dynamics). Top and bottom rows display results for random and peak sampling, respectively.

**Figure 15.**
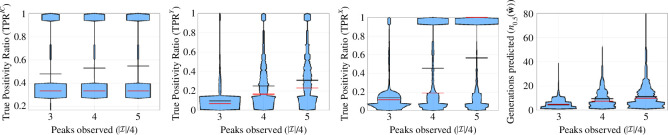
Violin plots comparing aggregate performance for peak sampling at 10% noise. Left to right: TPR${,}^{IC}$ (model recovery accuracy for the initial conditions map), TPR${,}^{Y}$ (model recovery accuracy for the continuous dynamics), TPR${,}^{X}$ (model recovery accuracy for the discrete dynamics). At 10% noise, it is clear that more samples are needed to achieve good long-term prediction. Sampling over five peaks yields correct identification of the discrete map in the majority of cases (third plot), which does not necessarily correspond to correct identification of the initial conditions map or the continuous dynamics (first and second plots). However, the predictive capability is still sufficient to accurately predict five generations ahead on average, even with only three peaks sampled (fourth plot), which may be sufficient for many applications. When five peaks are observed, 10 generations on average may be predicted.

In the low-data/high-noise limit, sparse regression is not expected to return perfect results, as the sparsity threshold adapts to the current noise level (see appendix B.2 and figure 8 for visualization of sparsity threshold selection under different noise levels). In figure 15 in the appendix, we include a treatment with 10% noise, displaying limitations in forward prediction accuracy. This is due to the unavoidable high coefficient errors imparted by large noise and low sampling rates, which limits the selectivity of sparse regression routines.^7^ In such cases, making use of the uncertainty as detailed in §4.5 is expected to faithfully quantify predictive loss.

In such data-poor regimes, there are several ways to improve results, such as limiting the number of terms in the model libraries to reduce the size of the model space, or structuring the candidate models such that certain terms are favoured (e.g. including them directly in the model *a priori*). For example, if intraspecific competition is known to be important, density-dependent survival or reproduction terms can easily be added to the models. In addition, some recent works have sought to overcome highly sparse samples of continuous-time dynamics using interpolation [49]. We leave a full investigation of techniques for handling such highly sparse regimes to future work.

We also note that measurement noise in the discrete (X) variables has not been considered, as it is more realistic that measurement errors are found in the continuous variables (which in reality are typically stochastic). A trade-off exists between noise in continuous and discrete variables, and indeed the present sampling regime is sensitive to noise in the discrete variables, due to the low number of observations and the fact that we are also estimating initial conditions for the continuous (Y) variables at each generation (noise in the discrete variables is amplified by the noise in the initial conditions). Data with |I|=12 generation pairs corresponds to linear systems with only 12 rows when identifying the initial conditions map and discrete dynamics, which is often insufficient to accurately select terms in the presence of noise in both X and Y. This could be alleviated by the introduction of a Bayesian framework to explicitly model the interplay between noise in X, noise in Y and model parameters, as in [8,29,30,92–95], and through the use of literature and experiment-derived prior knowledge as a way to regularize the estimation and provide more efficient inference. We defer the Bayesian extensions to future work.

## Figures and Tables

**Figure 9.**
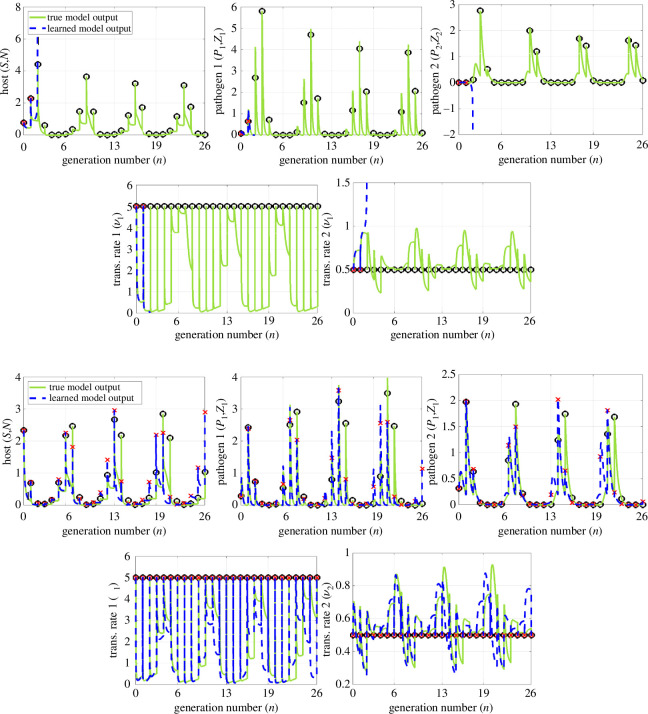
Two-pathogen example: 1% noise, in-sample and out-of-sample predictions. Top: ground truth data (green, black) used to learn the two-pathogen model in table 5 with WSINDy-Eco, as well as corresponding simulations (blue, red) from a representative model learned on the ground truth data with 1% noise added (similar to figure 1 in the main text). Bottom: simulations from the same learned model tested on an out-of-sample dataset (i.e. initialized at a new random initial condition).

**Figure 10.**
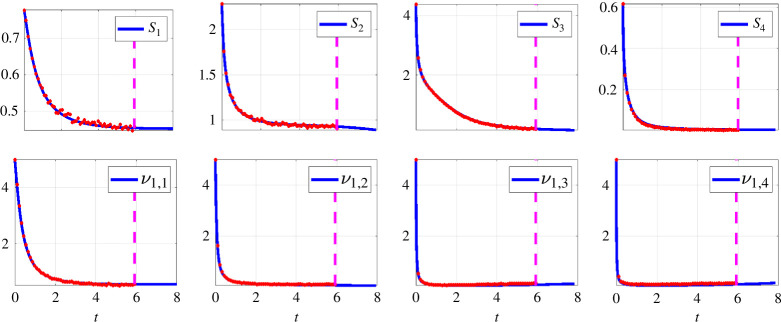
Two-pathogen example: sharp transition rate dynamics. Example data (red) and simulation (blue) used to obtain $S_{n}(T)$ and $\nu_{1,n}(T)$ for the first four generations (the vertical dashed magenta line indicates the maximum time observed, beyond which simulation from the learned model is used to obtain values at time T=8). As can be seen in the bottom row, the continuous $\nu_{1}$ dynamics are imperceptible at this sampling rate, leading to low recovery rates for the ${\dot{\nu }}_{1}$ equation (see figure 11). The data are the same as in figure 9.

**Figure 11.**
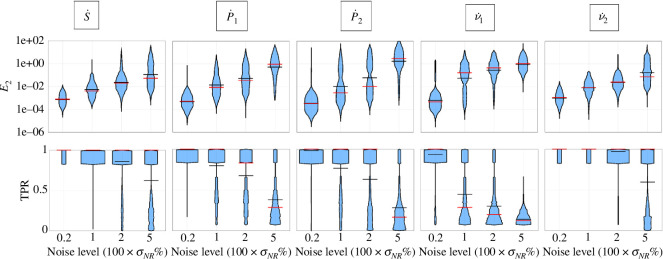
Aggregate performance of WSINDy-Eco in identifying continuous two-pathogen dynamics. Coefficient error ($E_{2}$, top) and model accuracy (TPR, bottom) of recovery of the continuous dynamics of the two-pathogen system using WSINDy-Eco.

**Figure 12.**
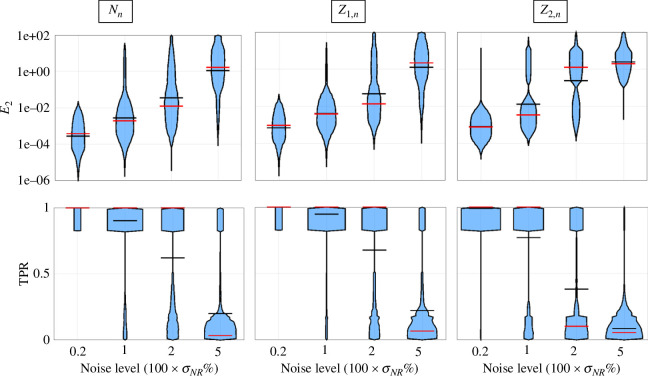
Aggregate performance of WSINDy-Eco in identifying discrete two-pathogen dynamics. Coefficient error ($E_{2}$, top) and model accuracy (TPR, bottom) of recovery of the discrete dynamics of the two-pathogen system using WSINDy-Eco.

**Figure 13.**
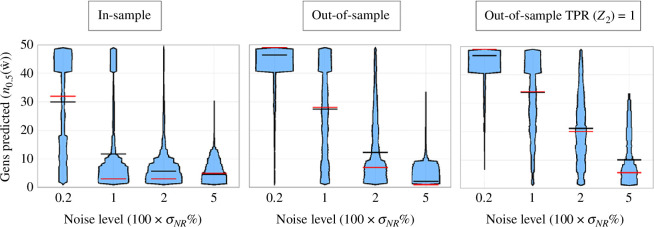
Aggregate performance of WSINDy-Eco in predicting two-pathogen dynamics. Number of generations predicted accurately ($n_{0.5}(\hat{𝐰})$) in applying WSINDy-Eco to the two-pathogen example using in-sample testing, out-of-sample testing and out-of-sample testing for learned models with correctly identified $Z_{2}$ dynamics (TPR = 1).

## Data Availability

All software used to generate the results in this work is available at the Github repository https://github.com/ MathBioCU/wsindy_eco and the permanent repository on Zenodo [96].

## Appendix A. Notation tables

Please see tables 2–4 for the overview of notation used in this paper.

**Table 2.** Notation concerning seasonal hybrid discrete-continuous dynamical systems.

category	symbol	definition	ref.
insect-pathogen case study	$\left(S_{n},P_{n}\right)$	host and pathogen density in continuous time	equation (2.1)
	$\left(N_{n},Z_{n}\right)$	host and pathogen density in discrete time	equation (2.2)
	{ν,V,μ,λ,ϕ,γ}	model parameters in host–pathogen system	§2.1
seasonal hybrid systems	$Y_{n}\in {\mathbb{R}}^{d}$	continuous variables	equation (2.5)
	$X_{n}\in {\mathbb{R}}^{D}$	discrete variables	equation (2.6)
	$t\in {\mathbb{R}}_{+}$	continuous time	equation (2.5)
	$T\in {\mathbb{R}}_{+}$	generation length	equation (2.6)
	$h:{\mathbb{R}}^{D}\to {\mathbb{R}}^{d}$	initial conditions map	equation (2.4)
	$g:{\mathbb{R}}^{d}\times {\mathbb{R}}^{D}\to {\mathbb{R}}^{d}$	continuous dynamics map	equation (2.5)
	$F:{\mathbb{R}}^{D}\times {\mathbb{R}}^{d}\to {\mathbb{R}}^{D}$	discrete dynamics map	equation (2.6)

**Table 3.** Notation concerning background on weak-form equation learning.

category	symbol	definition	ref.
equation learning	$y(t)\in {\mathbb{R}}^{d}$	state variables	equation (3.1)
	$y_{+}(t)$	temporal update of y	equation (3.1)
	$𝐭=(t_{1},\ldots ,t_{M})$	observed time points	§3.1
	$𝐘\in {\mathbb{R}}^{M\times d}$	observations of y at 𝐭	§3.1
	Δ	unit of discrete time	§3.1
	$𝕃=(f_{j}{)}_{j=1}^{J}$	model library	equation (3.2)
	$𝐖\in {\mathbb{R}}^{J\times d}$	model coefficients	equation (3.2)
weak formulation	X	function space	§3.1
	$X^{*}$	topological dual of X	§3.1
	⟨⋅,⋅⟩	duality pairing between X and $X^{*}$	§3.1
	$L_{2}$, $\ell_{2}$	square integrable functions	§3.1
	$\varphi \in X^{*}$	an element of $X^{*}$	equation (3.3)
	ϕ∈X	a representer test function for$\varphi \in X^{*}$	equation (3.4)
	$\Phi =(\phi_{1},\ldots ,\phi_{K})$	subset of K test functions	§3.1 WSINDy
	⟨⟨⋅,⋅⟩⟩	discretization for ⟨⋅,⋅,⟩ given Φ	§3.1 WSINDy
	δ	kronecker delta-function	§3.1 WSINDy
	$(𝐆,𝐁)\in {\mathbb{R}}^{K\times J}\times {\mathbb{R}}^{K\times d}$	weak-form linear system	§3.1 WSINDy
	${𝐓}_{+},{𝐓}_{-}$	forward/backward shift operators	§3.1 WSINDy
	$\hat{𝐖}\in {\mathbb{R}}^{J\times d}$	estimated model parameters	equation (3.8)

**Table 4.** Summary of notation pertaining to the MSTLS-WENDy and WSINDy-Eco algorithms.

category	symbol	definition	ref.
MSTLS-WENDy	𝐑	weak-form residual	§3.1 WENDy
	𝐫,𝐲,𝐰,𝐛	vectorized versions of 𝐑,𝐘,𝐖,𝐁	§3.1 WENDy
	D	abstract left-hand side operator	§3.1 WENDy
	Σ	covariance of data 𝐲	§3.1 WENDy
	𝐋(𝐰)	covariance factor	§3.1 WENDy
	δ𝐲	observed data minus truth	§3.1 WENDy
	${𝐋}^{\left(j\right)},j=0,\ldots ,Jd$	covariance factors	§3.1 WENDy
	𝐂(𝐰)	approximate covariance of 𝐫	§3.1 WENDy
	κ	sparsity thresholds	equation (3.8)
	$I_{ji}^{\kappa }$	non-uniform thresholding bounds	equation (3.9)
	${𝐰}^{\left(0\right)}$	initial guess for WENDy (typically the OLS solution)	§3.1 WENDy
	${𝐈}^{\left(d\right)}$	identity matrix in ${\mathbb{R}}^{d}$	§3.1 WENDy
	${𝐆}_{{,}_{⊗}}$	Kronecker matrix ${𝐈}^{\left(d\right)}⊗𝐆$ for regression in WENDy	§3.1 WENDy
	${𝕃}^{\left(\ell \right)}$	library at ℓth stage of MSTLS-WENDy	§3.1 MSTLS-
	${𝕃}_{+}$	library increment operator	equation (3.12)
	$\hat{𝐰}$	approximate (vectorized) coefficients	§3.1 MSTLS-
	$\hat{𝐂}$	approximate residual covariance	§3.1 MSTLS-
	$\hat{𝐒}$	approximate parameter covariance	§3.1 MSTLS-
	$\hat{𝐫}$	residual evaluated at approximate coefficients $\hat{𝐰}$	§3.1 MSTLS-
	$r^{\left(\ell \right)}$	mean-squared residual at ℓth stage	§3.1 MSTLS-
	${\hat{M}}^{\left(\ell \right)},{\hat{V}}^{\left(\ell \right)}$	approximate mean and variance of $r^{\left(\ell \right)}$	§3.1 MSTLS-
	Ψ	standard normal CDF	§3.1 MSTLS-
	c,tol	library incrementation confidence and tolerance	§3.1 MSTLS-
WSINDy-Eco	$I\subset \{0,\ldots ,N_{\max}\}$	set of observed generation pairs	§3.2
	$𝐗=\{(X_{n},X_{n+1}){\}}_{n\in I}$	observed discrete variables	§3.2
	$𝐘=\{Y_{n}({𝐭}_{n}){\}}_{n\in I}$	observed continuous variables and time points	§3.2
	$(\cdot {)}^{IC}$	reference to initial conditions map	§3.2
	$(\cdot {)}^{Y}$	reference to continuous dynamics	§3.2
	$(\cdot {)}^{X}$	reference to discrete dynamics	§3.2
	${\hat{𝐘}}_{T}$	estimated continuous dynamics at final time T	§3.2
	${\hat{\Sigma }}^{{\hat{𝐘}}_{T}}$	estimated covariance for ${\hat{𝐘}}_{T}$	§3.2
	$\left(u^{\left(s\right)},u^{\left(p\right)}\right)$	state and parametric variables	§3.2 Parametric
	$\left(\Sigma^{\left(s\right)},\Sigma^{\left(p\right)}\right)$	state and parametric covariances	§3.2 Parametric
	${𝕃}_{\left(s⊗p\right)}$	Kronecker parametric model library	§3.2 Parametric
	μ(t),σ(t)	mean and variance of log-normal r.v.	§4.1
	$\sigma_{NR}$	data noise level	§4.1
	$Y_{n}^{⋆}$	noise-free data	§4.1
	TPR	true positivity ratio	§4.2
	$E_{2}$	relative coefficient error	§4.2
	$\Delta_{n}$	relative forward simulation error	§4.2
	$n_{\text{tol}}(𝐰)$	forward simulation length	§4.2
	$P_{j}^{\left(\ell \right)}$	jth peak of the ℓth simulation	§4.3

## Appendix B. Supplementary info: section 3

### B.1. Algorithms

**MSTLS-WENDy**. Algorithm 2 contains pseudocode for the MSTLS-WENDy algorithm, a sparse regression routine for dynamical system identification which accounts for the errors-in-variables nature of the problem.

**MSTLS-WENDy-Par**. Algorithm 3 contains pseudocode for the MSTLS-WENDy-Par algorithm, which adapts MSTLS-WENDy to the setting of equations with both state and parametric variables.

**ForwardSim**. In the cases of interest, the continuous variable at time T, $Y_{n}(T)$ is not observed and must be simulated using the learned system. This is carried out for n∈I using Runge-Kutta 45 routine in Matlab (and easily parallelized over n∈I). This is detailed in algorithm 4, which includes a heuristic computation for the covariance ${\hat{\Sigma }}^{{\hat{𝐘}}_{T}}$ of the resulting estimates ${\hat{𝐘}}_{T}$.

### B.2. WSINDy-Eco hyperparameters

Throughout we set the WENDy iteration tolerance to $\tau =10^{-4}$, typically reached in under 10 iterations, and we use MaxIts=5. The library, test function and data covariance estimation choices are described below, together with a discussion about the sparsity parameters.

**Trial function libraries**. In this work, we let each library 𝕃 be of power–law type, with functions $f_{j}(u)=u_{1}^{p_{1}}\cdots u_{d}^{p_{d}}$ for $p_{i}\in \mathbb{R}$. These trial functions are chosen for their ability to model any dynamical system if $p_{i}$ is chosen large enough, as well as their computational efficiency, and their widespread appearance in models of natural phenomena. The parameter libraries ${𝕃}_{\left(p\right)}^{\text{IC}},{𝕃}_{\left(p\right)}^{X}$ are initialized to only contain the constant function {1}, while ${𝕃}_{\left(p\right)}^{Y}$ is initialized to contain the terms $\left\{1,N_{n}^{-V}\right\}$. They are then incremented by increasing the total polynomial degree by 1 in each incrementation step, up to a total degree of 4. The state libraries ${𝕃}_{\left(s\right)}^{Y},{𝕃}_{\left(s\right)}^{X}$ are fixed with total degrees 3 and 2, that is, one degree greater than that appearing in the true model (or what we think the true model is); in addition the term $S_{n}^{1+V}$ is added to ${𝕃}_{\left(s\right)}^{Y}$. Altogether this leads to a total of 562 possible terms across all equations in all sub-models.

**Test function basis**. For the continuous dynamics, we employ piece-wise polynomial test functions of the form

$$\phi_{k}(t)=\max{\left\{0,1-{\left(\frac{t-t_{k}}{m_{t}\Delta t}\right)}^{2}\right\}}^{\eta },$$

which are supported on the time intervals $\left(t_{k}-m_{t}\Delta t,t_{k}+m_{t}\Delta t\right)$, where Δt is the within-epidemic sampling rate and $m_{t}$ is the *test function radius*. The power η controls the highest order of differentiability, and thus the degree of accuracy of numerical integration in the absence of noise. The radius $m_{t}$ is largely responsible for variance reduction and mitigating high-frequency noise. The parameters $\eta ,m_{t}$ are chosen from the observed data 𝐘 using the method detailed in [11, appendix A], which interprets the weak form as an approximate low-pass filter.

**Data covariance estimation**. The covariance matrix $\Sigma^{𝐘}$ for the observed continuous data $𝐘=(Y_{n}({𝐭}_{n}){)}_{n\in I}$ is taken to be diagonal and is estimated by assuming that each value in the time series $Y_{n}(𝐭)$ has a constant variance $\sigma_{n}$, which we approximate using a high-order filter (see [97, appendix G]). The covariance matrix $\Sigma^{\text{IC}}$ for the initial condition data is then taken to be diagonal with variance $\sigma_{n}$ for $Y_{n}(0)$ and variance 0 for $X_{n}$. For the covariance matrix $\Sigma^{𝐗}$ of the data $\left(X_{n+1},X_{n},Y_{n}(T)\right)$, we assume that $Y_{n}(T)$ is random but $X_{n},X_{n+1}$ are non-random (have zero variance). We set the variance estimate for each $Y_{n}(T)$ to be the empirical variance of the observed data $Y_{n}({𝐭}_{n})$ around computed values ${\hat{Y}}_{n}({𝐭}_{n})$, as described in algorithm 4.

**Sparsity thresholds**. We use equally log-spaced candidate thresholds $\log_{10}\;\kappa =\text{ 𝚕𝚒𝚗𝚜𝚙𝚊𝚌𝚎}(-4,0,50)$, seen to work well in previous studies utilizing MSTLS [10,11]. The ultimate sparsity threshold κ used in the each final model is determined by the MSTLS loss function [11]. We note that there is a clear relationship between the selected threshold and the level of noise in the data, with higher noise levels corresponding to the need for (and the selection of) larger thresholds, and consequently less complex models. Figure 8 displays this trend for two exemplary cases, using peak sampling with three peaks at 1% and 5% noise, respectively. The selected threshold is seen to adapt to the noise level; however, at larger noise it may be challenging to select the correct model, as many models fare equally well according to the given loss (bottom middle plot).

## Appendix C. Supplementary info: section 4. Additional example: two-pathogen epizootic

We now present results after applying the algorithm to a challenging higher dimensional model as presented in [21], which can be viewed as an extension to the model we presented in the main text. The true model is now a two-pathogen epizootic/epidemic model, and is described by the following set of equations:

(C 1)
$$\begin{array}{r} \frac{\text{d}S}{\text{d}t}=-\nu_{1}P_{1}S-\nu_{2}P_{2}S, \end{array}$$

(C 2)
$$\begin{array}{r} \frac{\text{d}P_{1}}{\text{d}t}=\nu_{1}P_{1}S-\mu_{1}P_{1}, \end{array}$$

(C 3)
$$\begin{array}{r} \frac{\text{d}P_{2}}{\text{d}t}=\nu_{2}P_{2}S-\mu_{2}P_{2}, \end{array}$$

(C 4)
$$\begin{array}{r} \frac{\text{d}\nu_{1}}{\text{d}t}=-C_{1}^{2}P_{1}\nu_{1}^{2}-\rho C_{1}C_{2}P_{2}\nu_{1}\nu_{2}, \end{array}$$

(C 5)
$$\begin{array}{r} \frac{\text{d}\nu_{2}}{\text{d}t}=-C_{2}^{2}P_{2}\nu_{2}^{2}-\rho C_{1}C_{2}P_{1}\nu_{1}\nu_{2}. \end{array}$$

Building on the model in the main text, here S is the density of susceptible hosts and $P_{1}$ and $P_{2}$ are the respective densities of the two pathogens in the environment. For the model in the main text, strictly speaking the transmission rate changes over time, but the model is simple enough that it is possible to express the transmission rate in terms of the host density [19]. The two-pathogen model in contrast is sufficiently complicated that we must instead write down equations that track changes in the average transmission rates of the two pathogens $\nu_{1}$ and $\nu_{2}$.

Because hosts with higher infection risk become infected more rapidly, the rate of change of the transmission rate of a given pathogen declines as the density of that pathogen increases, at a rate that is higher if the transmission rate of the pathogen is higher, and if the host has higher variation $C_{i}$ with respect to that pathogen. In addition, however, the transmission rate of each pathogen changes because of infections with the other pathogen. The Pearson product–moment correlation ρ determines whether susceptibility to $P_{1}$ is positively or negatively correlated with susceptibility to $P_{2}$ (and vice versa).

As in the model in the main text, we use difference equations to extend the two-pathogen model to allow for multiple host generations. The model is as follows:

(C 6)
$$\begin{array}{r} N_{n+1}=\lambda N_{n}(1-I_{1}(N_{n},Z_{1,n},Z_{2,n})-I_{2}(N_{n},Z_{1,n},Z_{2,n})) \end{array},$$

(C 7)
$$\begin{array}{r} Z_{1,n}=\phi_{1}N_{n}I_{1}(N_{n},Z_{1,n},Z_{2,n}) \end{array},$$

(C 8)
$$\begin{array}{r} Z_{2,n}=\phi_{2}N_{n}I_{2}(N_{n},Z_{1,n}.Z_{2,n}). \end{array}$$

Here $N_{n}$, $Z_{1,n}$ and $Z_{2,n}$ are the densities of the host, pathogen 1 and pathogen 2, respectively, at the beginning of the epizootic in generation n. The symbol λ then represents host fecundity, while $\phi_{1}$ and $\phi_{2}$ represent the effective overwintering survival of each pathogen. The fraction of hosts infected by pathogens $P_{1}$ and $P_{2}$ in generation n are given by $I_{1}$ and $I_{2}$. Although not expressible in closed form, $I_{1}$ and $I_{2}$ may be represented using an additional set of ODEs,

(C 9)
$$\begin{array}{r} {\dot{I}}_{1}=\nu_{1}P_{1}S/N_{n} \end{array},$$

(C 10)
$$\begin{array}{r} {\dot{I}}_{2}=\nu_{2}P_{2}S/N_{n}. \end{array}$$

Further, $I_{2}$ may be deduced from $I_{1}$ and $S_{n}(T)$ using the total fraction I of hosts infected in generation n and the relation

$$I:=1-\frac{S_{n}(T)}{N_{n}}=I_{1}+I_{2}\;\;⟹\;\;I_{2}=1-\frac{S_{n}(T)}{N_{n}}-I_{1}.$$

Setting ${\tilde{I}}_{1,n}=N_{n}I_{1}$, this two-pathogen system can be written in the seasonal hybrid form (2.4)–(2.6) provided in table 5.

$$\left\{ \begin{array}{l} S_{n}(0)=w_{1}^{IC}N_{n}\\ P_{1,n}(0)=w_{2}^{IC}Z_{1,n}\\ P_{2,n}(0)=w_{3}^{IC}Z_{2,n}\\ \nu_{1,n}(0)=w_{4}^{IC}\\ \nu_{2,n}(0)=w_{5}^{IC}\\ {\tilde{I}}_{1,n}(0)=w_{6}^{IC} \end{array}\right.\;\left\{ \begin{array}{l} {\dot{S}}_{n}(t)=w_{1}^{Y}\nu_{1,n}(t)S_{n}(t)P_{1,n}(t)+w_{2}^{Y}\nu_{2,n}(t)S_{n}(t)P_{2,n}(t)\\ {\dot{P}}_{1,n}(t)=w_{3}^{Y}\nu_{1,n}(t)S_{n}(t)P_{1,n}(t)+w_{4}^{Y}P_{1,n}(t)\\ {\dot{P}}_{2,n}(t)=w_{5}^{Y}\nu_{2,n}(t)S_{n}(t)P_{2,n}(t)+w_{6}^{Y}P_{2,n}(t)\\ {\dot{\nu }}_{1,n}(t)=w_{7}^{Y}\nu_{1,n}^{2}(t)P_{1,n}(t)+w_{8}^{Y}\nu_{1,n}(t)\nu_{2,n}(t)P_{2,n}(t)\\ {\dot{\nu }}_{2,n}(t)=w_{9}^{Y}\nu_{2,n}^{2}(t)P_{2,n}(t)+w_{10}^{Y}\nu_{1,n}(t)\nu_{2,n}(t)P_{1,n}(t)\\ {\dot{\tilde{I}}}_{1,n}(t)=w_{11}^{Y}\nu_{1,n}(t)S_{n}(t)P_{1,n}(t) \end{array}\right.\;\left\{ \begin{array}{l} N_{n+1}=w_{1}^{X}S_{n}(T)\\ Z_{1,n+1}=w_{2}^{X}{\tilde{I}}_{1,n}(T)\\ Z_{2,n+1}=w_{3}^{X}N_{n}+w_{4}^{X}S_{n}(T)\\ +\;w_{5}^{X}{\tilde{I}}_{1,n}(T) \end{array}\right.$$

**Table 5.** The host–pathogen system with two pathogens considered in numerical experiments, written in the form of (2.4)–(2.6). The coefficients $\left\{{𝐰}^{IC},{𝐰}^{Y},{𝐰}^{X}\right\}$ used to generate synthetic data are given in the first row of tables, and the initial conditions are given in the bottom table. The coefficients correspond to parameter values $\left\{\mu_{1},\mu_{2},C_{1},C_{2},\rho ,\lambda ,\phi_{1},\phi_{2}\}=\{0.99,0.32,2.06,0.68,-0.5,5,2,2\right\}$ following guidelines in [21] for generating stable cycles. The initial conditions model an invasion by $P_{2}$ into a stable one-pathogen system consisting of $\left(S,P_{1}\right)$.

Eq	terms	${𝐰}^{IC}$
$S_{n}(0)$	$\left[N_{n}\right]$	1.0
$P_{1,n}(0)$	$\left[Z_{1,n}\right]$	1.0
$P_{2,n}(0)$	$\left[Z_{2,n}\right]$	1.0
$\nu_{1,n}(0)$	[1]	5.0
$\nu_{2,n}(0)$	[1]	0.5
${\tilde{I}}_{1,n}(0)$	[1]	0.0

Eq	terms	${𝐰}^{Y}$
${\dot{S}}_{n}$	$\left[\nu_{1,n}S_{n}P_{1,n}],[\nu_{2,n}S_{n}P_{2,n}\right]$	(-1.0, -1.0)
${\dot{P}}_{1,n}$	$\left[\nu_{1,n}S_{n}P_{1,n}],[P_{1,n}\right]$	(1.0, -0.99)
${\dot{P}}_{1,n}$	$\left[\nu_{2,n}S_{n}P_{2,n}],[P_{2,n}\right]$	(1.0, -0.32)
${\dot{\nu }}_{1,n}$	$\left[P_{1,n}\nu_{1,n}^{2}],[P_{2,n}\nu_{1,n}\nu_{2,n}\right]$	(−4.2436, 0.7)
${\dot{\nu }}_{2,n}$	$\left[P_{1,n}\nu_{1,n}^{2}],[P_{2,n}\nu_{1,n}\nu_{2,n}\right]$	(−0.4624, 0.7)
${\dot{\tilde{I}}}_{1,n}$	$\left[\nu_{1,n}S_{n}P_{1,n}\right]$	1.0

Eq	terms	${𝐰}^{X}$
$N_{n+1}$	$\left[S_{n}(T)\right]$	5
$Z_{1,n+1}$	$\left[{\tilde{I}}_{1,n}(T)\right]$	2
$Z_{2,n+1}$	$[N_{n},\;S_{n}(T)],$ $\left[{\tilde{I}}_{1,n}\left(T\right)\right]$	(2, -2, -2)

$N_{0}$	$Z_{1,0}$	$Z_{2,0}$
0.198	0.0402	0.0001

### C.1. Data generation and WSINDy-Eco hyperparameters

We consider the case study in [21] of a pathogen invasion (by $P_{2}$) into a stable one-pathogen system consisting of $\left(S,P_{1}\right)$. In [21], it was demonstrated that the second pathogen will survive, and stable cycles will emerge, if its mean susceptibility $\nu_{2}$ and coefficient of variation $C_{2}$ are sufficiently smaller than that of $P_{1}$. To generate these dynamics, we fix the model parameters at the values in table 5. This results in the dynamics in figure 9 (top), which depicts time traces for the five continuous variables $S,P_{1},P_{2},\nu_{1},\nu_{2}$ in green overlaying the discrete variables $N_{n},Z_{1},Z_{2}$ in black. Pathogen 2 rapidly invades and beyond generation 20, a stable cycle of 7 emerges (the blue curves and red x’s in this figure, as well as the entire second set of five plots, are discussed below).

Due to the high dimensionality of the two pathogen system, it is necessary to restrict the library 𝕃 from all possible monomial combinations of the variables up to a fixed total degree as in the main text. Following standard models in ecology, we make the simplifying assumption that each library term in the equations for $\dot{S},{\dot{P}}_{1},{\dot{P}}_{2},{\dot{\nu }}_{1}$ and ${\dot{\nu }}_{2}$ depends on the state variable on the left-hand side, $S,P_{1},P_{2},\nu_{1}$, or $\nu_{2}$, respectively. In addition, we exclude terms that depend on ${\tilde{I}}_{1}$ and assume that the terms in the equation for ${\dot{\tilde{I}}}_{1}$ are known up to their coefficients. The rest of the WSINDy-Eco hyperparameters are the same as in the main text. For sampling, we examine only the five-peak sampling case from in the main text (figure 10).

### C.2. Results

Figures 11–13 display the results of 100 random trials applying WSINDy-Eco to data from the two-pathogen system for each of the noise levels $\sigma_{NR}\in \{0.002,0.01,0.02,0.05\}$. We first note that the initial conditions map (top left set of equations in table 5) was recovered accurately in each case, with correct terms (TPR = 1) and relative coefficient errors ($E_{2}$) below the data noise level $\sigma_{NR}$.

Similar to the results in the main text, WSINDy-Eco recovers each of the continuous dynamics ($\dot{S},{\dot{P}}_{1},{\dot{P}}_{2},{\dot{\nu }}_{1},{\dot{\nu }}_{2}$), as well as the discrete dynamics ($N,Z_{1},Z_{2}$), robustly at low noise levels (e.g. 0.2%, see figures 11 and 12). At 1% noise, recovery of the equation for ${\dot{\nu }}_{1}$ begins to fail (figure 11, fourth column), while the remaining equations are robustly identified, with median TPR equal to 1. This is because $\nu_{1}$ rapidly reaches steady state in the first few time steps, leaving very little information about its dynamics (see figure 10, bottom row). At 2% noise, $Z_{2}$ also begins to lack robust recovery, and at 5% noise most equations are challenging to identify. It should be noted that the discrete dynamics are identified from a highly underdetermined system of just 60 rows and 210 columns of possible terms.

Figure 13 displays the prediction accuracy of the learned models as measured by $n_{0.5}(\hat{𝐰})$ using *in-sample* testing and *out-of-sample* testing. In-sample testing (left plot of figure 13) refers to simulating the learned model from the same initial conditions $X_{0}=(N_{0},Z_{1,0},Z_{2,0})$ as the training data. In a slight abuse of terminology, out-of-sample testing (middle plot of figure 13) refers to simulating the learned model on five random initial conditions drawn from a uniform distribution with mean X and variance $0.25X^{2}$, where X is the average of the respective $X\in \{N,Z_{1},Z_{2}\}$ values observed in the training data.

As can be seen in figure 13, in-sample testing is highly sensitive to noise. This is due to the low initial pathogen 2 density ($P_{2,0}$) leading to many learned models predicting that $P_{2}$ will die out in the first few generations. For example, for data with 1% noise, we record 75% of learned models, each trained on data with a different independent noise sequence, predicting that $P_{2}$ will die out in the first five generations. This is exactly the scenario in figure 9 (top five plots), in which the learned model from data with 1% noise predicts that $P_{2}$ dies out in the third generation (as shown, without halting conditions that learned model eventually leads to negative $P_{2}$ values). This sensitivity manifests as a numerical instability in the discrete update equation for $Z_{2}$, but can also be readily interpreted from an ecological perspective. Coefficient errors as low as 1% in the $Z_{2}$ update, even with correct equation identification (TPR = 1), can result in non-positive initial $P_{2,n+1}(0)$ values when $P_{2,n}(T)$ is sufficiently low, from which $P_{2}$ density cannot recover. Put another way, for sufficiently low density, the second pathogen is not able to survive the winter.

For comparison, out-of-sample testing performs surprisingly well, with only 12% of models predicting that $P_{2}$ dies out in the first five generations at 1% noise. The bottom five plots of figure 9 show an out-of-sample test that uses the same learned model as the in-sample test in the plots above it. Despite misidentifying the equation for $\nu_{1}$, the model predicts the next four host outbreaks to within a year on this out-of-sample dataset. From this we conclude that the ‘pathogen 2 invasion’ case examined here is particularly challenging to model, but it nevertheless provides rich enough data to WSINDy-Eco to learn models that generalize well to a variety of cases.

In the rightmost plot of figure 13, motivated by the challenges associated with identifying the $Z_{2}$ dynamics as exhibited in figure 12, we further examine the out-of-sample testing case but for learned models that identified the $Z_{2}$ equation correctly with TPR = 1. The learned models exhibit significantly increased prediction lengths across each noise level. This result, together with the sensitivities of in-sample testing discussed in the previous paragraphs, emphasizes the importance of quantifying the effective over-wintering survival rate of competing pathogen strains; reassuringly, the necessary experiments are logistically challenging but feasible [21]. Moreover, correct identification of the $Z_{2}$ dynamics does not correlate with correct identification of the dynamics of ${\dot{\nu }}_{1}$ which is the other equation that turned out to lack robust recovery (figure 11 column 4). We therefore conjecture that this model is not particularly sensitive to the ${\dot{\nu }}_{1}$ dynamics.

In summary, in this additional analysis we have shown that the algorithm presented in the main text can be used to infer higher dimensional and thus harder problems. The problem that we have studied is characterized by fast changing state dynamics which makes it more challenging to model and learn in the case in which there are only a few terms. Indeed, the magnitude of the problem is even more severe for other methods, for example parameter-inference-only methods with known model structure, for which sophisticated statistical parameter estimation techniques such as MCMC tend to be very slow for anything but very simple versions of the models that we use here [21].

## Appendix D. Supplementary info: section 4(e)

The Akaike information criterion (AIC) is defined in general by [6,84]

(D 1)
$$\text{AIC}=2k-2\log(\hat{L}),$$

where k is the number of non-zero entries of the model parameter vector $\hat{𝐰}$ and L=L(𝐰) is a suitable likelihood function, and $\hat{L}=L(\hat{𝐰})$ is its maximum, attained at the parameters $\hat{𝐰}$. In our case, the negative log-likelihood function is simply given by the sum of each generalized least-squares residual,

(D 2)
$$\begin{array}{rl} -\log(L(\hat{w}))=\frac{1}{2}[(G^{\text{IC}} & {\hat{w}}^{\text{IC}}-b^{\text{IC}}{)}^{T}({\hat{C}}^{\text{IC}}{)}^{-1}(G^{\text{IC}}{\hat{w}}^{\text{IC}}-b^{\text{IC}})\\ & +(G^{Y}{\hat{w}}^{Y}-b^{Y}{)}^{T}({\hat{C}}^{Y}{)}^{-1}(G^{Y}{\hat{w}}^{Y}-b^{Y})\\ & \;+(G^{X}{\hat{w}}^{X}-b^{X}{)}^{T}({\hat{C}}^{X}{)}^{-1}(G^{X}{\hat{w}}^{X}-b^{X})] \end{array}$$

with superscripts {IC,X,Y} denoting the submodel which the quantity corresponds to. Similarly, the number of non-zero model parameters is $k=\|\hat{𝐰}{\|}_{0}=\|{\hat{𝐰}}^{\text{IC}}{\|}_{0}+\|{\hat{𝐰}}^{Y}{\|}_{0}+\|{\hat{𝐰}}^{X}{\|}_{0}$.

## Appendix E. Supplementary info: section 4(f)

Figure 14 complements figure 7 with additional performance metrics including information about the initial conditions map and the continuous dynamics. The trends are analogous, with peak sampling offering a clear advantage, and requiring fewer samples to achieve accurate model recovery. In figure 15, we report results for peak sampling under 10% noise, showing a breakdown in the long-term predictive capabilities of the learned model. However, at this high noise level, learned models are still able to predict on average 5–10 generations in the future, which may be sufficient for many applications including the spongy moth one where insect outbreaks tend to occur every 9–12 years.

^1^
In forest insect populations, densities during troughs in the outbreak cycles are so low that finding any insects at all is often a problem [20], and so sampling typically focuses on the years immediately preceding and following peak population sizes [50,51].
^2^
Coefficient of variation is defined as the ratio of the standard deviation to the mean, and serves as a measure of dispersion, or heterogeneity, for a distribution. For a Gamma (α,β) density, it is equal to $\alpha^{-1/2}.$
^3^
Although this approach need not be restricted to time-dependent phenomena.
^4^
We will use subscripts, such as $W_{ji}$, the (i,j)th entry of matrix W, as indices. We will also avoid changing the bold font style, keeping $W_{ji}$ instead of e.g. $w_{ji}$.
^5^
In what follows, quantities representing estimates of the ground truth will be denoted with hats.
^6^
This holds for large Kd, where K is the number of test functions and d is the number of equations. In the examples below, Kd=84|I| for the continuous dynamics and Kd=2|I| for the discrete and initial conditions maps, where |I| is the number of observed generations
^7^
We remark that in [9], it is reported that 10% noise and coarse sampling (e.g. 64 points in time) leads to 10% relative errors in the coefficients on average, which is expected to lead to errors in the sparse identification.
